# Modulation of the endogenous omega-3 fatty acid and oxylipin profile *in vivo*—A comparison of the *fat-1* transgenic mouse with C57BL/6 wildtype mice on an omega-3 fatty acid enriched diet

**DOI:** 10.1371/journal.pone.0184470

**Published:** 2017-09-08

**Authors:** Annika I. Ostermann, Patrick Waindok, Moritz J. Schmidt, Cheng-Ying Chiu, Christopher Smyl, Nadine Rohwer, Karsten-H. Weylandt, Nils Helge Schebb

**Affiliations:** 1 Institute for Food Toxicology, University of Veterinary Medicine Hannover, Hannover, Germany; 2 Food Chemistry, Faculty of Mathematics and Natural Sciences, University of Wuppertal, Wuppertal, Germany; 3 Institute for Parasitology, Centre for Infection Medicine, University of Veterinary Medicine Hannover, Hannover, Germany; 4 Medical Department, Division of Hepatology and Gastroenterology (including Metabolic Diseases), Charité University Medicine Berlin, Campus Virchow Klinikum, Berlin, Germany; 5 Experimental and Clinical Research Centre, Charité University Medicine, Campus Buch, Berlin, Germany; 6 Medical Department, Division of Gastroenterology, Oncology, Hematology, Rheumatology and Diabetes, Ruppiner Kliniken, Brandenburg Medical School, Neuruppin, Germany; Laval University, CANADA

## Abstract

Dietary intervention and genetic *fat-1* mice are two models for the investigation of effects associated with omega-3 polyunsaturated fatty acids (n3-PUFA). In order to assess their power to modulate the fatty acid and oxylipin pattern, we thoroughly compared *fat-1* and wild-type C57BL/6 mice on a sunflower oil diet with wild-type mice on the same diet enriched with 1% EPA and 1% DHA for 0, 7, 14, 30 and 45 days. Feeding led after 14–30 days to a high steady state of n3-PUFA in all tissues at the expense of n6-PUFAs. Levels of n3-PUFA achieved by feeding were higher compared to *fat-1* mice, particularly for EPA (max. 1.7% in whole blood of *fat-1* vs. 7.8% following feeding). Changes in PUFAs were reflected in most oxylipins in plasma, brain and colon: Compared to wild-type mice on a standard diet, arachidonic acid metabolites were overall decreased while EPA and DHA oxylipins increased with feeding more than in *fat-1* mice. In plasma of n3-PUFA fed animals, EPA and DHA metabolites from the lipoxygenase and cytochrome P450 pathways dominated over ARA derived counterparts.*Fat-1* mice show n3-PUFA level which can be reached by dietary interventions, supporting the applicability of this model in n3-PUFA research. However, for specific questions, e.g. the role of EPA derived mediators or concentration dependent effects of (individual) PUFA, feeding studies are necessary.

## Introduction

It has long been suggested that dietary intake of long-chain omega-3 polyunsaturated fatty acids (n3-PUFA), especially of eicosapentaenoic acid (C20:5 n3, EPA) and docosahexaenoic acid (C22:6 n3, DHA) is associated with beneficial health effects [1, 2]. Strong evidence exists for an improvement of cardiometabolic health by lowering blood trigylceride levels and cardiovascular outcomes, such as sudden cardiac death [2]. Furthermore, anti-inflammatory [1, 2] and anti-angiogenic [3, 4] effects have been described. Part of the effects might be explained by direct physiological actions of n3-PUFA. They have been shown to act directly on membrane ion channels, or to reduce expression of inflammatory genes via nuclear factor-kappaB (NFκB), e.g. by interacting with peroxisome proliferator-activated receptor gamma (PPARγ) [1, 2]. Moreover, n3-PUFA serve as substrates in the arachidonic acid (C20:4 n6, ARA) cascade. In this signaling cascade, ARA is converted via three enzymatic pathways and autoxidation to oxidative metabolites, called oxylipins, several of which are biologically highly active: (I) Cyclooxygenase (COX) conversion of ARA yields series-2 prostanoids, like the potent prostaglandin (PG) E_2_ which is involved in the regulation of pain, fever and inflammation or thromboxane (Tx) A_2_ which is involved in platelet aggregation [5–7]. (II) Lipoxygenase (LOX) action on ARA leads to multiple biologically active classes of lipid mediators via hydroperoxy intermediates, such as leukotrienes (LT), e.g. LTB_4_, involved in the chemotaxis of neutrophils, lipoxins with anti-inflammatory properties or hydroxy-FA (OH-FA) [5–7]. (III) Finally, cytochrome P450 (CYP) enzymes can convert ARA to OH- and epoxy-FA (Ep-FA). For instance, ω-hydrolase activity of CYP enzymes can yield the vasoconstrictory 20-hydroxyeicosatetraenoic acid (20-HETE) formed by members of the CYP4A or 4F family [5, 7, 8]. Conversion of ARA by CYP2C and 2J, e.g., leads to vasodilatory, anti-inflammatory, analgesic and angiogenic acting Ep-FA [5, 7–10] which are further metabolized to less potent dihydroxy-FA (DiH-FA) by the soluble epoxide hydrolase [5, 7, 11]. The effect of n3-PUFA on this important signaling cascade is multifaceted. On the one hand, by competing with ARA for conversion, the formation of potent ARA derived mediators, such as pro-inflammatory PGE_2_ and LTB_4_ is reduced, while their EPA derived counterparts, PGE_3_ and LTB_5_ have been shown to be less potent [1, 7]. On the other hand, enzymatic conversion of EPA and DHA can yield highly potent lipid mediators: CYP catalyzed epoxidation leads e.g. to anti-arrhythmic acting 17(18)-epoxy eicosatetraenoic acid (EpETE) from EPA and 19(20)-epoxy docosapentaenoic acid (EpDPE) from DHA [12]. Interestingly, while these Ep-FA share the anti-inflammatory action of the corresponding ARA oxylipins [3], 19(20)-EpDPE has been shown to inhibit angiogenesis in contrast to ARA derived Ep-FA [4]. Moreover, multiple hydroxylation leads to highly potent, specialized pro-resolving lipid mediators (SPM) such as resolvins and protectins [13, 14]. A comprehensive overview about the ARA cascade can be found in recent reviews, e.g. [5, 7, 15].

Regarding the clinical relevance of n3-PUFA in different diseases the results of epidemiological and intervention studies are conflicting [1, 2, 16]. Moreover, molecular modes of action for individual effects of n3-PUFA have not been fully unveiled, and dose dependencies remain largely unclear [1, 2]. In order to address these questions, appropriate experimental models allowing a well-defined modulation of the endogenous n3-PUFA and oxylipin profile are required.

In humans and other mammals EPA and DHA can be synthesized endogenously by combined elongation, desaturation and β-oxidation reactions from the essential n3-PUFA alpha linolenic acid (C18:3 n3, ALA) [17, 18]. However, conversion rates are low on a diet rich in linoleic acid (C18:2 n6, LA), as it is the case for a typical western diet (soy, corn and sunflower oil based) [17]. In humans and other mammals, endogenous supply of EPA and DHA thus relies on the dietary intake. In *in vivo* studies, animal diets are often enriched with EPA and DHA containing oils to modulate the endogenous n3-PUFA profile. This approach has been used in different disease models for the investigation of n3-PUFA associated biology, e.g. in inflammatory diseases, such as colon inflammation [19, 20], arthritis [21, 22], hypertension [23], liver injury [24] or Parkinson’s Disease [25].

Another approach to investigate physiological effects of elevated endogenous n3-PUFA concentrations is the use of the *fat-1* transgenic mouse model. The DNA of these mice has been edited to carry the *fat-1* gene of the nematode *Caenorhabditis elegans* encoding an n3 fatty acid desaturase catalyzing the conversion of n6 to n3 fatty acids [26]. This leads to a decreased endogenous n6/n3-ratio in *fat-1* mice fed with a standard n6-PUFA rich diet compared to wild type (WT) animals, e.g. from 46.6 (WT) to 2.9 (*fat-1*) in erythrocytes [26]. Therefore, *fat-1* mice have been used in many studies for the investigation of n3-PUFA associated effects, e.g. in inflammation, including colitis [27, 28], hepatitis [29], pancreatitis [30], different types of cancer, such as liver [31], colitis-associated colon cancer [32, 33] and melanoma [34] as well as Parkinson’s Disease [35] or chemically induced diabetes [36].

In most studies using *fat-1* mice, selected FA and/or (variations of) the n6/n3-PUFA ratio in tissues are used to describe of the endogenous n3-PUFA status [27–37]. Only little attention has been paid to the modulation of n3- and n6-PUFA oxylipins in *fat-1* compared to wild type mice. In disease models a focus was set on selected oxylipins, such as PGE and/or PGD from ARA and/or EPA [27, 28, 32, 34, 36], SPMs [28], precursor thereof [31] and few others [28, 32, 36]. A comprehensive set of free oxylipins in *fat-1* mice has only been described in plasma [38] and total (free and esterified) OH-FA have been described in plasma and tissues [37]. Almost no data is available on the differences in fatty acids and oxylipins in *fat-1* versus WT mice after dietary supplementation with n3-PUFA. The only available study compares the effects of nine weeks of feeding on selected oxylipins and fatty acids in kidney tissue [39]. Therefore, in the present study we thoroughly investigated the modulation of both, the total fatty acid and oxylipin profile in *fat-1* vs WT mice on a standard, sunflower oil based diet and the same diet enriched with n3-PUFA (1% EPA and 1% DHA). Not only is a comprehensive set of tissues and blood (including plasma and blood cells) analyzed, we also show the time course of effects occurring on a diet enriched with n3-PUFA over a feeding period of 7–45 days. This study provides fundamental insights on the breadth of effects on the lipidome caused by the insertion of the *fat-1* gene into the murine DNA in the context of a diet high in n6-PUFA compared to an n3-PUFA dietary intervention as well as the time dependency of nutrition induced changes.

## Materials and methods

### Chemicals

Acetic acid and methanol (Optima LC/MS Grade) as well as acetonitrile (HPLC-MS grade) were obtained from Fisher Scientific (Schwerte, Germany) and ammonium acetate (p.a.) was purchased from Merck (Darmstadt, Germany). Methyl *tert*-butyl ether and *n*-hexane (HPLC grade) were obtained from Carl Roth (Karlsruhe, Germany). Methyl tricosanoate (FAME C23:0) was obtained from Santa Cruz Biotechnology (Heidelberg, Germany). Oxylipin and deuterated oxylipin standards were purchased from Cayman Chemicals (local distributor: Biomol, Hamburg, Germany). Further oxylipin standards (Epoxy octadecadienoic acids (EpODEs) and dihydroxy octadecadienoic acids (DiHODEs)) were a kind gift from the laboratory of Bruce Hammock (UC Davis, CA, USA). Ethyl acetate, methyl formate and all other chemicals were purchased from Sigma Aldrich (Taufkirchen, Germany).

### Feeding experiment

Animals were cared for in accordance with the institution’s guidelines for experimental animals based on EU Directive 2010/63/EU. All experiments were carried out and use of the animals was registered and approved by the Landesamt für Gesundheit und Soziales Berlin (Reg No.: T0025/13). Pellets for the feeding experiment were based on a standard experimental diet from ssniff (product number: E15051; ssniff Spezialdiäten GmbH, Soest, Germany) with 10% fat. The fat used for the standard diet (STD) was refined sunflower oil (Henry Lamotte Oils, Bremen, Germany) enriched with 0.2% (*w/w*) tocopherol mix (Covi-Ox T 70 EU, BASF, Ludwigshafen). The n3-PUFA rich diet (STD+n3) was the same diet containing 1% EPA and 1% DHA as ethyl esters (10% each in fat). Ethyl ester are generated during the purification and concentration of fish oil and thus represent together with the re-esterified triglycerides the most important class of supplementation products [40]. The fat content, peroxide value and the fatty acid composition of the experimental diets can be found in the supplementary information (S1 Table).

Heterozygous transgenic *fat-1* mice were generated as described [26] and phenotyping (ratio of n6/n3-PUFA) from tails was carried out using gas chromatography. For the feeding experiment female C57BL/6 WT and *fat-1* mice of 9–10 weeks of age were used (n = 6 per feeding group). Before the experiment, mice were kept on a diet with 3.3% fat (1.8% LA, 0.23% ALA in the diet, S2 Table) with water and food supply ad libitum. The chow was stored in plastic bags containing an oxygen absorber at -20°C. During the whole feeding experiment fresh chow was provided every 2–3 days. WT mice were kept on the experimental diets for 7, 14, 30 and 45 days. In order to compare the fatty acid profile in *fat-1* mice to WT mice fed with an n3-PUFA enriched diet (maximum modulation of the fatty acid profile in blood and tissues following 30 days of feeding; see below), *fat-1* mice were kept on the standard sunflower diet for 30 days. One group of WT and *fat-1* animals was sacrificed on day 0. Animals were killed by cervical dislocation and organs (liver, kidney, spleen, brain and colon) as well as blood (by cardiac puncture) were collected. 10 μL of whole blood were directly diluted with 50 μL of deionized water. For plasma and blood cell generation, blood was directly centrifuged (800 x *g*, 10 min, 4°C). Plasma was collected and blood cells were washed once with phosphate buffered saline (containing 1.5 mg/mL ethylenediaminetetraacetic acid (EDTA)) and reconstituted to the original blood volume in phosphate buffered saline. All samples were stored at -80°C until further analysis.

### Fatty acid analysis

Fatty acid composition was analyzed in all collected blood fractions (60 μL diluted whole blood, 50 μL plasma and 100 μL reconstituted blood cells) and tissues (30–35 mg) as described [41]. Briefly, blood and tissues were extracted with methanol/ methyl *tert*-butyl ether (1:2, *v/v*) and derivatized to fatty acid methyl esters (FAME) with methanolic hydrogen chloride (acetylchloride in methanol (1:10, *v/v*)) before analysis was carried out using gas chromatography with flame ionization detection (GC-FID). For the calculation of the relative pattern and absolute fatty acid concentrations response factors were used [41, 42]. Results are presented as mean ± standard error of the mean (SEM).

### Oxylipin analysis

Extraction and analysis of oxylipins from plasma (200 μL) and colon (50±5 mg) was carried out as described [43]. Brain (50±5 mg) was homogenized following addition of internal standards and antioxidant solution [43, 44] in 750 μL ethyl acetate and 500 μL water (pH 6) in a ball mill using two 3 mm metal beads (25 Hz, 5 min, Retsch, Haan, Germany). Following homogenization and centrifugation (20 000 x *g*, 5 min, 4°C), the organic phase was collected and the sample extracted with another 750 μL ethyl acetate. The combined organic phases were evaporated using a vacuum centrifuge (Christ, Osterode am Harz, Germany) and the dried lipid extract was reconstituted in 300 μL methanol. All samples were diluted to 6 mL with water and acidified with acetic acid (to pH 3) directly before extraction on C18 cartridges (500 mg, Macherey-Nagel, Düren, Germany). Methyl formate was used for elution. Oxylipins were quantified by liquid chromatography-mass spectrometry (LC-MS) as described [43, 44]. Hemolytic plasma samples and samples with high TxB_2_ and 12-HETE—indicating improper anticoagulation—were excluded from analysis. Results are presented as mean ± SEM.

### Statistical analysis

Statistical analyses were performed as indicated using GraphPad Prism version 7.00 for Windows, GraphPad Software, La Jolla California USA, www.graphpad.com.

## Results

### Behavior and bodyweight

During the experiment, no differences in animal behavior were observed and similar body weights between the feeding groups (17.9–21.7 g, supplementary information, S1 Fig) indicated no differences in feeding behavior.

### Fatty acid profile

**Fig 1:**
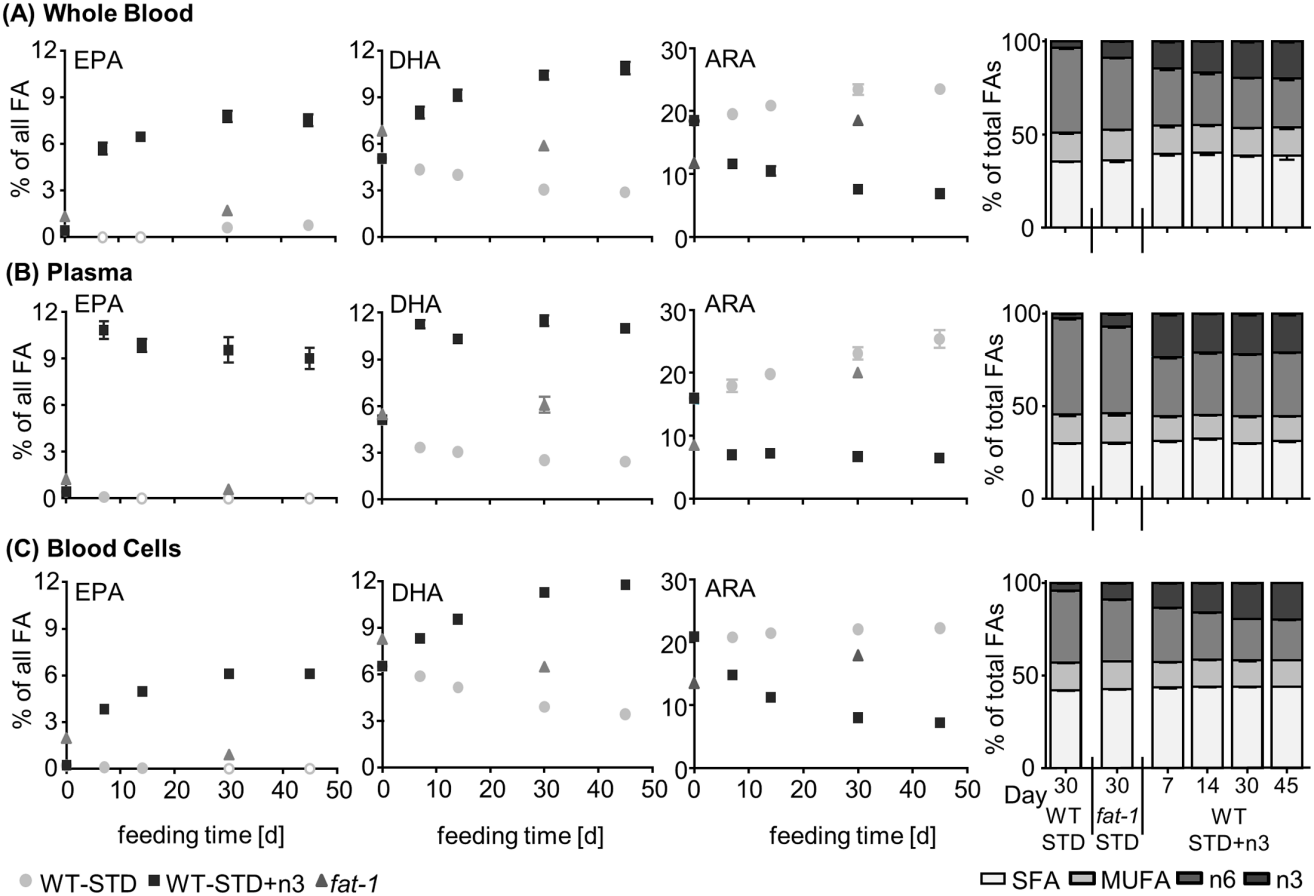
Fatty acid profile in blood. Shown are relative amounts of EPA, DHA and ARA as well as the relative distribution of n3- and n6-PUFA, MUFA and SFA in transgenic *fat-1* mice and wild type animals (WT-STD) on a sunflower oil based diet, as well as in wild type mice on the same diet enriched with EPA and DHA (WT-STD+n3) during the course of the feeding period (45 days) in (A) whole blood, (B) plasma and (C) blood cells. Analytes that were below the limit of quantification are marked with a white filling in the diagram. Results of the statistical analyses for selected fatty acids during the course of the feeding with the n3-PUFA enriched diet as well as for WT and *fat-1* mice after 30 days on the experimental diets are shown in S6+S7 Tables.

Fig 1 and Table 1 show the relative pattern of selected FA as well as the FA profile grouped as saturated fatty acids (SFA), monounsaturated fatty acids (MUFA) and n6- and n3-polyunsaturated fatty acids (PUFA) during the course of the feeding time. The full FA profile of blood and tissues can be found in the SI (S3A+S3B Table).

**Table 1:** Fatty acid profile in tissues. Shown are relative amounts of EPA, DHA and ARA as well as the sum of n6-PUFA, n3-PUFA, MUFA and SFA in liver, kidney, spleen, colon and brain tissue in WT (WT-STD) and *fat-1* mice after 30 days on a standard sunflower oil based diet and in WT mice on the same diet enriched with EPA and DHA (WT-STD+n3) during the course of the feeding period (day 7–45). Results of the statistical analyses for selected fatty acids during the course of the feeding with the n3-PUFA enriched diet as well as for WT and *fat-1* mice after 30 days on the experimental diets are shown in S6+S7 Tables.

		EPA	DHA	ARA	n6-PUFA	n3-PUFA	MUFA	SFA
**Liver**								
WT-STD	Day 30	<LOQ	3.7 ± 0.2	15.9 ± 0.9	39.5 ± 0.8	3.8 ± 0.2	24 ± 2	33 ±1
*fat-1*	Day 30	0.16 ± 0.02	6.8 ± 0.5	13.2 ± 0.7	36.7 ± 0.6	7.2 ± 0.5	24 ± 2	32 ± 1
WT-STD+n3	Day 7	6.1 ± 0.4	14.7 ± 0.3	7.8 ± 0.2	26.8 ± 0.6	22.3 ± 0.6	13.5 ± 0.5	37.4 ± 0.3
	Day 14	4.4 ± 0.2	11.1 ± 0.4	5.9 ± 0.4	27 ± 1	17.0 ± 0.6	21 ± 1	35.1 ± 0.8
	Day 30	5.4 ± 0.4	14.1 ± 0.8	5.1 ± 0.2	26.3 ± 0.5	21 ± 1	19 ± 2	34.7 ± 0.5
	Day 45	4.2 ± 0.5	12.5 ± 0.5	6.1 ± 0.2	29 ± 1	18.1 ± 0.9	18.9 ± 0.6	34.4 ± 0.5
**Kidney**								
WT-STD	Day 30	0.015 ± 0.002	7.4 ± 0.6	22 ± 2	38.8 ± 0.7	7.7 ± 0.6	16 ± 2	38.0 ± 0.6
*fat-1*	Day 30	1.01 ± 0.07	12.1 ± 0.3	20.3 ± 0.6	35.5 ± 0.5	13.9 ± 0.4	12.1 ± 0.5	38.5 ± 0.3
WT-STD+n3	Day 7	4.6 ± 0.1	14.9 ± 0.7	12.9 ± 0.8	27.8 ± 0.3	20.6 ± 0.8	13 ± 1	39.1 ± 0.3
	Day 14	5.2 ± 0.2	16.3 ± 0.7	10.9 ± 0.6	25.7 ± 0.2	22.6 ± 0.8	13 ± 1	38.7 ± 0.2
	Day 30	6.3 ± 0.3	18.0 ± 0.5	10.3 ± 0.4	24.6 ± 0.2	25.5 ± 0.6	11.0 ± 0.7	39.0 ± 0.2
	Day 45	6.2 ± 0.2	16.5 ± 0.7	9.5 ± 0.5	25.2 ± 0.3	23.9 ± 0.9	13 ± 1	38.1 ± 0.4
**Spleen**								
WT-STD	Day 30	0.035 ± 0.007	2.6 ± 0.1	20.4 ± 0.8	39 ± 1	3.0 ± 0.1	16 ± 2	42.8 ± 0.5
*fat-1*	Day 30	1.6 ± 0.2	4.9 ± 0.6	14 ± 1	31.2 ± 0.2	9 ± 1	18 ± 3	41 ± 1
WT-STD+n3	Day 7	3.58 ± 0.07	9.8 ± 0.3	10.5 ± 0.5	25.7 ± 0.4	17.8 ± 0.4	13.3 ± 0.9	43.2 ± 0.2
	Day 14	4.0 ± 0.1	10.3 ± 0.2	8.3 ± 0.4	23.4 ± 0.4	18.7 ± 0.4	14.8 ± 0.7	43.0 ± 0.3
	Day 30	4.3 ± 0.1	11.2 ± 0.2	7.7 ± 0.2	23.2 ± 0.2	20.1 ± 0.5	13.1 ± 0.7	43.6 ± 0.3
	Day 45	3.8 ± 0.2	10.5 ± 0.6	6.9 ± 0.5	23.6 ± 0.2	19 ± 1	16 ± 2	42.1 ± 0.9
**Brain**								
WT-STD	Day 30	<LOQ	17.1 ± 0.3	11.3 ± 0.2	15.5 ± 0.3	17.2 ± 0.3	20.5 ± 0.6	46.8 ± 0.2
*fat-1*	Day 30	0.066 ± 0.005	16.3 ± 0.9	9.8 ± 0.8	14.0 ± 0.9	16.5 ± 0.9	25 ± 3	45 ± 1
WT-STD+n3	Day 7	0.15 ± 0.02	16.7 ± 0.4	10.3 ± 0.3	14.3 ± 0.4	17.1 ± 0.4	23 ± 1	46.0 ± 0.4
	Day 14	0.14 ± 0.01	17.4 ± 0.3	10.6 ± 0.2	14.6 ± 0.3	17.9 ± 0.3	20.9 ± 0.7	46.6 ± 0.3
	Day 30	0.18 ± 0.01	19.0 ± 0.3	9.5 ± 0.2	13.2 ± 0.2	19.6 ± 0.3	21.2 ± 0.5	46.0 ± 0.2
	Day 45	0.16 ± 0.01	17.4 ± 0.5	8.8 ± 0.2	15.8 ± 0.4	15.8 ± 0.6	20 ± 1	48.7 ± 0.3
**Colon**								
WT-STD	Day 30	0.048 ± 0.010	0.73 ± 0.13	5.7 ± 1.0	35.5 ± 0.8	0.96 ± 0.15	35± 1	29.0 ± 0.8
*fat-1*	Day 30	0.65 ± 0.22	1.5 ± 0.5	4.7 ± 1.4	32 ± 1	2.9 ± 0.8	35 ± 2	31 ± 1
WT-STD+n3	Day 7	1.8 ± 0.3	3.5 ± 0.4	2.8 ± 0.8	28.8 ± 0.6	6.7 ± 0.7	32 ± 2	32.9 ± 0.8
	Day 14	2.8 ± 0.4	5.0 ± 0.5	3.6 ± 0.6	26.0 ± 0.6	9.2 ± 0.8	30 ± 1	34.6 ± 0.7
	Day 30	2.0 ± 0.2	4.5 ± 0.3	1.8 ± 0.4	27.5 ± 0.6	7.7 ± 0.5	30.5 ± 0.9	34.2 ± 0.6
	Day 45	3.0 ± 0.4	5.7 ± 0.2	3.5 ± 0.4	27.4 ± 0.5	10.2 ± 0.6	27.4 ± 0.8	35.0 ± 0.3

Feeding of a diet enriched in EPA and DHA (1% EPA and 1% DHA as ethyl ester, WT-STD+n3) led to a time dependent increase in the relative and absolute concentrations of n3-PUFA, particularly EPA, DHA and n3 docosapentaenoic acid (22:5n3, n3-DPA), in blood and all investigated tissues (Fig 1, Table 1, S2 Fig, S3 Table). The feeding time necessary to reach a maximal increase was tissue dependent; however, 14–30 days were sufficient to reach maximum level in all tissues and blood (as % of total FA, Fig 1, Table 1, S3 Table). The overall FA pattern was changed least in brain (Table 1, S2D Fig).

After 30 days on the STD+n3 diet, EPA ranged in blood and tissues from 0.18–9.5% (brain/plasma [min./max.]) and DHA levels ranged from 4.5–19% (colon/brain, Fig 1, Table 1). While the absolute increase in EPA and DHA compared to baseline was in a similar range for both FA (S3B Table), individual relative differences, expressed as mean %difference [(c(FA)_WT-STD+n3, D30_—c(FA)_WT, D0_)/c(FA)_WT, D0_*100], were remarkably higher for EPA compared to DHA due to low baseline levels of EPA (S3 Fig). In all groups (WT mice on the standard, sunflower oil based diet (WT-STD), WT-STD+n3, and *fat-1*) EPA level in blood and tissues were lower as compared to DHA. It should be noted, that in response to n3-PUFA feeding, SFA and MUFA changed only slightly (however, significantly for MUFA in many tissues and blood), while n6-PUFA levels, particularly ARA, were significantly decreased (Fig 1, Table 1, S2 and S3 Figs, S6 Table).

*Fat-1* mice fed 30 days with a standard, sunflower oil based diet showed higher levels of EPA, n3-DPA and DHA in comparison to WT animals on the same diet (Fig 1, Table 1, S3B Table) reaching statistical significance for EPA and DHA in many tissues (S7 Table). However, compared to WT-STD+n3 mice, level of EPA and DHA were significantly lower in *fat-1* mice (p<0.0001 for all tissues and blood, except DHA in brain, Fig 1, Table 1, S7 Table). Particularly, levels of EPA were low (0.066–1.7%; brain/whole blood; Fig 1, Table 1) which is also reflected in the high %difference of EPA between both groups (S4 Fig). Relative differences in DHA and n3-DPA were more moderate in most tissues and blood between WT-STD+n3 and *fat-1* mice (S4 Fig).

**Fig 2:**
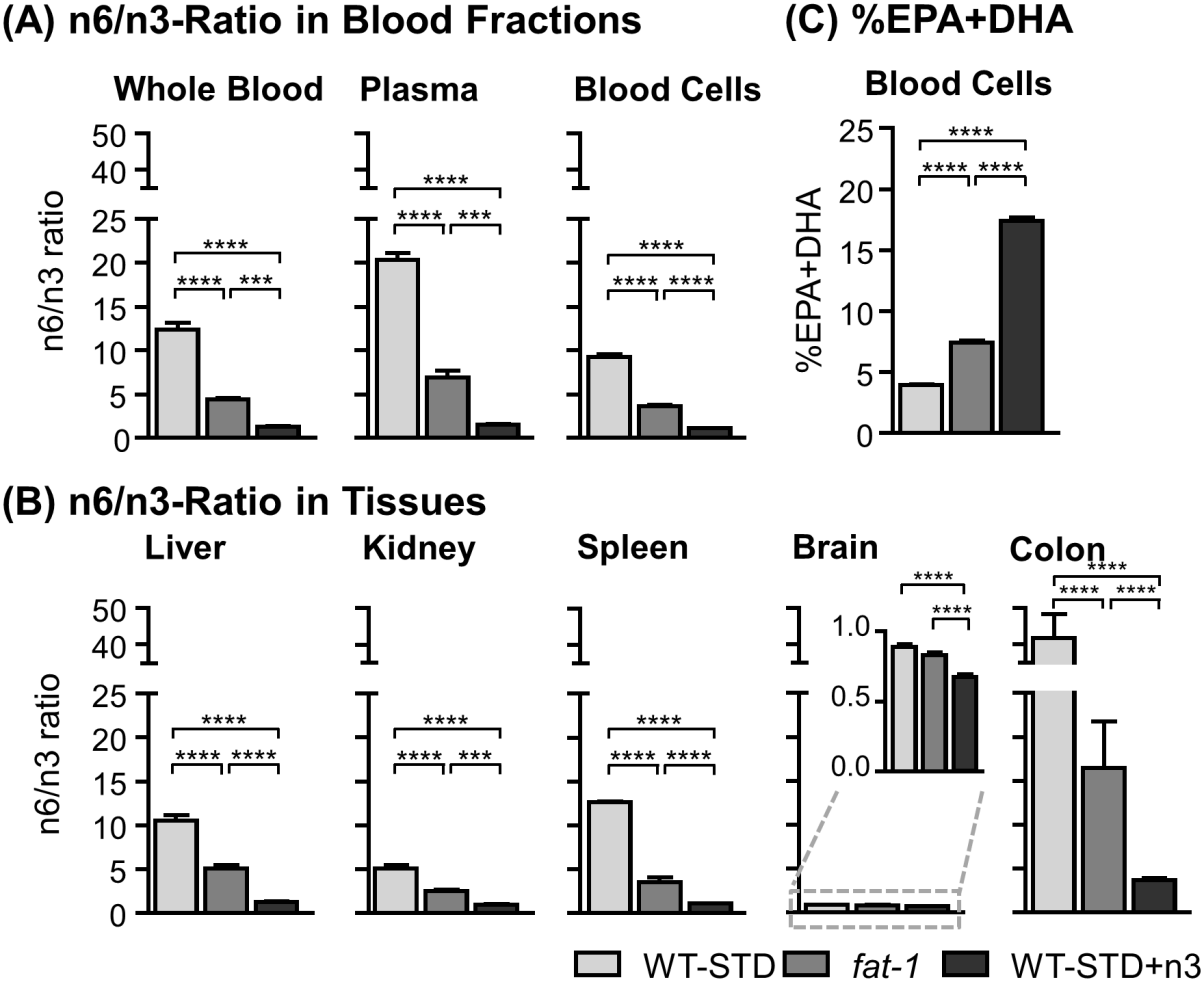
n6/n3 ratio in blood as well as tissues and %EPA+DHA in blood cells. Shown is the n6/n3 ratio in blood (A) and in tissues (B), as well as %EPA+DHA in blood cells (C) in transgenic *fat-1* mice and wild type animals (WT-STD) on a standard sunflower oil based diet, as well as in wild type mice on the same diet enriched with EPA and DHA (WT-STD+n3) after 30 days of feeding. The n6/n3 ratio was calculated as Ʃ%(C18:2 n6, C18:3 n6, C20:3 n6, C20:4 n6, C22:4n6)/ Ʃ%(C18:3 n3, C20:5 n3, C22:5 n3, C22:6 n3). Statistical differences were determined using one-way ANOVA followed by Tukey’s post test (*** p<0.001, **** p<0.0001).

The n6/n3 ratio in blood and tissues as well as the sum of %EPA and %DHA (%EPA+DHA) in blood cells, a modification of the omega-3 index [45], as marker for the endogenous n3-PUFA status are presented in Fig 2 for the different groups after 30 days on the experimental diets. Data on blood and tissues in all groups can be found in the SI (S5 Fig, S4 Table). The n6/n3 ratio in WT-STD+n3 mice was below 2 in all tissues and blood (except colon with 3.6) and 2.5–6.9 in *fat-1* mice (except brain and colon with 0.83 and 16 respectively), being significantly lower than in WT-STD mice with n6/n3 ratios of 5.1–20 (except brain and colon with 0.89 and 41, Fig 2A+2B). %EPA+DHA in blood cells was significantly higher (p<0.0001) in WT-STD+n3 (17.4±0.2%) and *fat-1* (7.4±0.2%) in comparison to WT-STD (3.9±0.1%, Fig 2C).

### Oxylipin pattern

**Fig 3:**
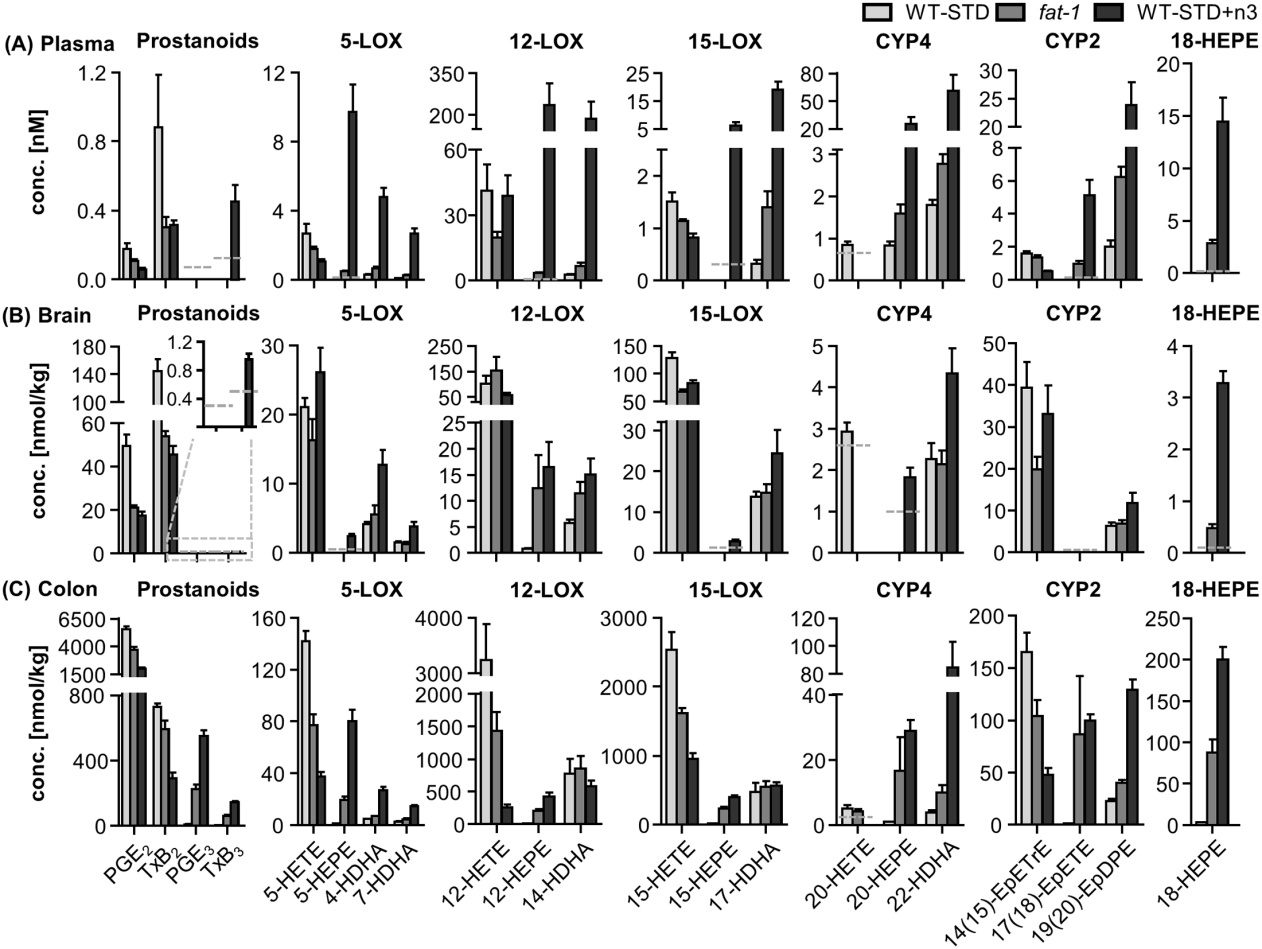
Concentrations of oxylipins. Presented are concentrations of selected prostanoids, 5-LOX, 12-LOX, 15-LOX, CYP4 and CYP2 products of ARA, EPA and DHA as well as 18-HEPE in (A) plasma, (B) brain and (C) colon in transgenic *fat-1* mice and wild type animals (WT-STD) on a sunflower oil based diet, as well as in wild type mice on the same diet enriched with EPA and DHA (WT-STD+n3) after 30 days of feeding. The lower limit of quantification (LLOQ) for the analyte is indicated in case it was not exceeded in >50% of the samples per group. Results of the statistical analyses for the comparison of oxylipins between the feeding groups after 30 days on the experimental diets are shown in S8 Table.

Oxylipins were analyzed in selected tissues, i.e. plasma, colon and brain. Concentrations of all oxylipins covered by the LC-MS method in all feeding groups in plasma, colon and brain are presented in S5 Table. Since a steady state in the nutrition induced changes in fatty acids (see above) was reached after 30 days, the differences between the groups are highlighted for this time point in Fig 3 for selected oxylipins from EPA, DHA and ARA.

Feeding of a diet enriched with n3-PUFA (1% EPA and 1% DHA as ethyl ester) led to high changes in the oxylipin pattern of plasma, brain and colon, especially in the first seven days of feeding (S5 Table). Due to low basal concentrations of EPA metabolites, their relative increase was overall higher compared to DHA metabolites (exemplary shown for 30 days of feeding in SI S6 Fig) while absolute increases in EPA and DHA metabolites were similar (except in brain, S5 Table). Reductions of the circulating ARA eicosanoids were less consistent compared to trends in the FA while ARA eicosanoids in colon and brain were uniformly decreased (S6 Fig).

**Fig 4:**
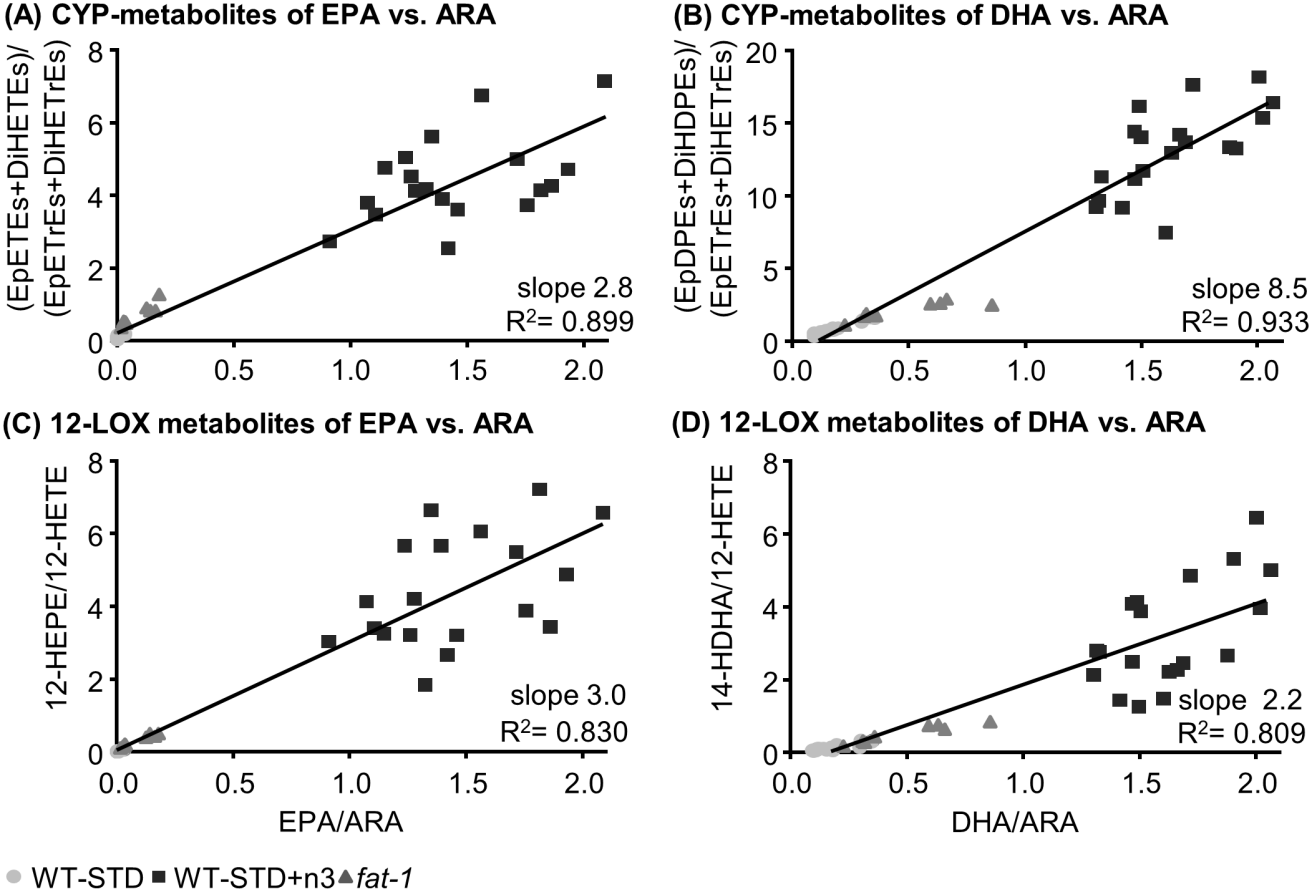
Correlation between plasma n3-PUFA/n6-PUFA oxylipins and the ratio of their precursor fatty acids. The ratio of CYP metabolites (sum of epoxy-FA and dihydroxy-FA) from EPA **(A)** and DHA **(B)** to the respective ARA metabolites are plotted against the ratio of their precursor PUFA. In panel **C-D** the same correlation is shown for selected 12-LOX metabolites (12-HETE and 14-HDHA). The slope of the linear regression and the correlation coefficient were calculated based on all feeding groups of the experiment.

After 30 days on the standard sunflower oil based diet, concentrations of EPA and DHA metabolites in plasma of *fat-1* mice were elevated, yet not significantly, compared to WT-STD mice (Fig 3, S8A Table). However, reflecting changes in FA, concentrations of EPA and DHA oxylipins in *fat-1* mice were significantly lower compared to WT-STD+n3 (Fig 3, S8A Table). Particularly, concentrations of oxylipins formed in the LOX and CYP pathway were high, dominating the oxylipin profile in plasma of WT-STD+n3 mice, while COX metabolites were barely altered. Interestingly, the sum of plasma oxylipins was highly elevated in WT-STD+n3 compared to *fat-1* and WT-STD (S7I Fig). Comparing the ratio of precursor PUFA and their oxidative products in all feeding groups, an almost linear correlation (R^2^>0.8) resulted for CYP and LOX metabolites (Fig 4). The slope of >2 indicates that moderate changes in the concentrations of EPA or DHA led to a more pronounced change in the concentrations of their oxylipins.

In colon and brain tissue both, *fat-1* and WT-STD+n3 mice showed a shift in the absolute and relative pattern towards n3-PUFA derived oxylipins after 30 days on the experimental diets in comparison to WT-STD mice (Fig 3, S7II Fig). As in plasma, WT-STD+n3 mice showed higher concentrations of n3-PUFA oxylipins compared to *fat-1*. However, differences between both groups were less pronounced, yet statistically significant in colon for many analytes, compared to plasma (Fig 3, S7I and S8 Figs, S8B+S8C Table). Additionally, in colon and brain tissue the sum of all oxylipins was decreased (S7I Fig). Although DHA in brain tissue showed minor differences between the feeding groups after 30 days on the experimental diets, DHA derived docosanoids were uniformly higher in WT-STD+n3 and *fat-1* mice as compared to WT-STD mice (Fig 3B), reaching statistical significance for some HDHAs (S8C Table). As for EPA, concentrations of EPA-eicosanoids in brain were low.

The increase in n3-PUFAs observed in plasma, brain and colon of WT-STD+n3 and *fat-1* compared to WT-STD was accompanied by a decrease in most ARA derived eicosanoids (Fig 3, S7I Fig, S5 Table). Similar to the higher level of n3-PUFA oxylipins in WT-STD+n3 compared to *fat-1*, concentrations of ARA metabolites in plasma and colon were lower in WT-STD+n3 than in *fat-1*, reaching statistical significance for many eicosanoids in colon. In brain, ARA eicosanoid levels in both groups were similar.

## Discussion

Feeding of n3-PUFAs EPA and DHA with the diet led to significantly higher concentrations of respective fatty acids in blood and tissues compared to WT mice on a standard sunflower diet as reported earlier in rodents [12, 19–22]. In men, similar observations were made following EPA+DHA supplementation, however, changes were more moderate caused by lower supplementation levels of n3-PUFA [46–52]. After 14–30 days of feeding a sunflower oil based diet enriched with 1% EPA and 1% DHA, levels of both FA reached a maximum steady state. This data suggests that studies aiming to investigate the effect of n3-PUFA need to implement a pre-feeding period of at least 14–30 days in order to maximally modulate the fatty acid profile. *Fat-1* mice showed significantly lower concentrations of n3-PUFA as compared to supplemented WT mice which was most pronounced for EPA, being 2.7–34 fold lower in blood and tissues, while DHA concentrations were at most threefold lower at maximum steady state at 30 days and thereafter (Fig 1, Table 1). This can be explained by the genetic background of these animals. In the *fat-1* mouse model the *fat-1* gene of the roundworm *C*. *elegans* was introduced into the DNA of C57BL/6 wildtype mice. As a consequence, these animals are able to biosynthesize n3- from n6-PUFA [26]. In *C*. *elegans*, different n6-PUFA, such as LA, C20:3 n6 and ARA are converted by the n3 desaturase, encoded by the *fat-1* gene, resulting in ALA, C20:4 n3 and EPA, respectively [53]. The roundworm lacks further elongase activity. Therefore, the biosynthetic fatty acid pathway stops at EPA, being the most abundant PUFA in the worm [53]. By contrast, in mammals EPA can be further elongated to n3-DPA which in turn can be converted via C24:5 n3 to DHA [17, 18]. These reactions occur at high rates, e.g. 63% (EPA to n3-DPA) and 37% (n3-DPA to DHA) in humans [54]. EPA biosynthesis in mammals from the essential ALA, however, is low—caused by the rate limiting desaturation of ALA to C18:4 n3 in combination with high dietary LA and low ALA consumption [19, 20]. This results in low endogenous EPA levels compared to DHA, being <0.05% in WT mice on a standard sunflower oil based diet in most tissues and blood (Fig 1, Table 1) or two- to tenfold lower than DHA in blood of non-supplemented humans [46, 47, 49–52].

Due to n3 desaturase activity in *fat-1* mice, endogenous EPA formation is higher compared to WT mice. However, further metabolism by mammalian enzymes again results in high DHA concentrations compared to EPA, e.g. 6.8% (DHA) vs. 0.16% (EPA) in liver, which is consistent with previous results [29]. Thus, n3 desaturase activity led to high DHA levels in *fat-1* while EPA levels were in the low range.

Levels of intermediary formed n3-DPA were in the same range as EPA levels in *fat-1* mice, supporting higher conversion rates of EPA to n3-DPA than of n3-DPA to DHA as observed in humans [54]. This finding is also supported by levels of n3-DPA in STD fed WT mice, which were higher compared to EPA levels in all investigated tissues and blood cells. However, it should be noted, that n3-DPA might also be formed in the process of DHA retroconversion: Following a single dose of 3 mg [^13^C]22:6-triacylglycerol to male rats (300 g), retroconversion was found to be 9% of the total plasma [^13^C]22:6 n3 (estimated by [^13^C]22:5 n3+[^13^C]20:5 n3 in plasma lipids) [55].

The n6/n3 ratio, a frequently used marker to describe the endogenous n3-PUFA status [19, 25, 28, 29, 32, 34, 56] was significantly reduced in *fat-1* compared to WT animals on a standard diet. Although calculated slightly different, the observed n6/n3 ratios were comparable, however, a little higher than ratios observed by Kang et al. [26]. Feeding of WT animals with a diet enriched with 1% EPA and 1% DHA led to significantly lower n6/n3 ratios. It remains to be determined if this is also associated with a higher degree of protection. In a model of Parkinson’s disease this seems to be the case: *Fat-1* mice did not show a neuroprotective effect [35], while n3-PUFA supplementation did [25]. The lack of efficiency in the *fat-1* mouse model might be due to a lower modulation of the endogenous n3 and n6 PUFA profile compared to supplementation, as discussed by Bousquet et al. [35].

The %EPA+DHA in blood cells for the description of the endogenous n3-PUFA status is a modification of the omega-3 index which is discussed as a risk factor for cardiovascular diseases in humans [45, 57]. In *fat-1* mice, %EPA+DHA in blood cells was 7.4±0.2%. Translating from mouse to man, these levels were in the range of an omega-3 index that has been shown to be correlated with a lower cardiovascular risk, e.g. for mortality from coronary heart disease (omega-3 index >8%, [45, 57]). %EPA+DHA in blood cells of *fat-1* mice was also comparable to the omega-3 index observed in healthy volunteers following supplementation (0.46–1.6 mg/d EPA and 0.38–1.1 g/d DHA for up to 12 weeks) which ranged from 8.4–11% (calculated from the means presented for EPA and DHA as %of total FA [46, 49, 50]). However, the ratio of DHA to EPA in *fat-1* mice was 7 while in humans after supplementation this ratio was on average two [46, 49, 50]. Thus, individual level of EPA and DHA were differently modulated in *fat-1* mice compared to n3-PUFA supplementation with almost equal amounts of EPA and DHA. Keeping in mind that EPA and DHA effects might be different (e.g. EpDPEs were more effective in reducing pain than EpETEs in a model of pain associated with inflammation [58]), care must be taken when directly transferring results obtained from the *fat-1* mouse model to humans. In response to higher endogenous level of n3-PUFA the share of n6-PUFA, such as ARA and related FA, was decreased which is consistent with previous findings in n3-PUFA fed animals on a dietary background high in LA [12, 21, 22] and *fat-1* mice [27–29, 31–36]. Thus, EPA and DHA supplementation directly led to a notable displacement of ARA, which is a common explanation for their anti-inflammatory action [1–3]. This theory is based on the assumption that most ARA derived oxylipins act pro-inflammatory and that n3-PUFA compete for conversion by the same enzymes yielding, e.g., less potent, EPA derived PGE_3_ or LTB_5_ [1, 7]. However, it should be noted, that many n3-PUFA derived oxylipins also possess anti-inflammatory properties [3, 7]. A pro-inflammatory phenotype might thus also result from a lack of n3-PUFA oxylipins. Nevertheless, our study supports a replacement of ARA by n3-PUFA on the level of oxylipins, reflecting the changes observed for PUFA (Fig 3): While oxylipin levels in WT animals on a standard diet—in line with the high ARA level—were dominated by ARA derived oxylipins, the pattern shifted to n3-PUFA derived ones in *fat-1* and n3-PUFA fed animals. This is consistent with previous results, showing similar trends for free oxylipins [38] and esterified OH-FA [37] in *fat-1* mice as well as for esterified CYP metabolites in plasma and tissues of rodents [12]. It should be noted that in general the modulation of the oxylipin pattern was more pronounced for n3-supplemented than for *fat-1* mice compared to WT mice on the standard diet. As a result, n3-PUFA feeding led overall to higher concentrations of n3-PUFA derived oxylipins and lower ARA derived eicosanoids (except in brain) compared to *fat-1* mice.

As shown in Fig 3 for exemplary oxylipins, the product patterns of the LOX (5, 12 and 15) and CYP (hydroxylation and epoxygenation) pathways in plasma after n3-PUFA feeding were dominated by EPA and DHA oxylipins. This is somewhat remarkable, since ARA remained a dominating PUFA (6.7% ARA vs. 9.5% EPA vs. 11% DHA in plasma of WT-STD+n3 mice, Fig 1) and indicates a preferred formation of n3-PUFA oxylipins over ARA derived ones. The ratio of substrates and products for CYP and LOX (Fig 4) found in this study suggests that a preference of the enzymes could explain part of the effect. However, the moderate preference of, e.g., epoxygenating and hydroxylating CYP enzymes for DHA or EPA over ARA (ARA:EPA:DHA 1:4:1.5 for CYP2J2) [12, 59] alone seems not to sufficiently explain the massive difference observed in the overall pattern of oxylipins in plasma. Interestingly, in tissues, the dominance of LOX and CYP derived n3-PUFA oxylipins was less pronounced and concentrations were mostly in a similar range as ARA derived eicosanoids which indicates a tissue specific regulation. Nevertheless, our findings once more show that a moderate shift in the fatty acid pattern causes a pronounced increase in their oxidation products.

In contrast, only a slight shift in ARA derived eicosanoids to n3-PUFA oxylipins was observed for COX products in plasma and tissues. While absolute concentrations of COX derived ARA metabolites were similarly decreased as LOX and CYP products, EPA metabolite concentrations in *fat-1* and n3-PUFA fed mice were still very low compared to their ARA derived counterparts. This can be explained by the low conversion rate of n3-PUFA by COX [60] leading on the one hand to low EPA-product formation and on the other hand to inhibition of ARA conversion by COXs.

It is interesting that compared to plasma and brain the highest concentrations of oxylipins from all three branches of the ARA cascade were found in colon, although the share of EPA, DHA and ARA among all FA in colon was low compared to other tissues and blood. Particularly COX metabolites were found in high concentrations, indicating an important role of these lipid mediators in homeostasis. Distinct differences in the oxylipin pattern were found between *fat-1* and WT mice on the STD diet in colon. While relative changes in ARA and DHA metabolites were moderate, EPA metabolites from all enzymatic pathways were massively increased in *fat-1* compared to WT-STD mice. This high increase may in part explain the effectiveness of the *fat-1* model in colitis and colitis-associated colon cancer [27, 28, 32, 33]. While specialized pro-resolving mediators (SPM) derived from EPA were not found in colon tissue of *fat-1* mice, 18-HEPE for example, as anti-inflammatory pathway indicator and precursor for E series resolvins [61] with unclear formation pathway, was highly increased.

In brain, only slight modulations in the fatty acid profile were found between the groups. Differences in oxylipins, however, were pronounced. This may have resulted from residual blood in the tissue, although highest care was taken during sample preparation. Nonetheless, the pronounced effect on brain oxylipin levels by n3-PUFA warrants further investigation.

Overall, the fatty acid and oxylipin pattern in *fat-1* mice and n3 supplemented mice were modulated to higher concentrations of n3-PUFA and their metabolites in blood and tissues compared to WT mice on a standard sunflower diet. In general, the modulation in *fat-1* mice was lower compared to n3-PUFA supplementation. The applicability of the *fat-1* mouse model to investigate n3-PUFA associated effects, however, has been demonstrated in various disease models, e.g. colon inflammation or hepatitis [27–34, 36]. Since levels of EPA+DHA in blood cells of *fat-1* mice were comparable to humans after supplementation, this model mimics n3-PUFA concentrations readily achievable with dietary supplementation. However, levels of the individual FA, particularly EPA were different, which might result in different physiological effects. An advantage of the *fat-1* mouse model in comparison to feeding of n3-PUFA is the possibility of using one standard diet for the experimental groups. Therefore, confounding factors which might be introduced by the use of different experimental diets [56] or degradation of oxidation prone PUFA to potentially bioactive compounds [62] are reduced. However, for several questions, this model might not be suitable, particularly for the investigation of concentration dependent effects or the optimization of the dietary n6/n3 ratio needed for protection against diseases [63]. Here, feeding studies using defined concentrations of n3-PUFA in the diet in combination with an effective feeding regime are the most suitable approach. Another drawback of the *fat-1* mouse model is that a discrimination between individual effects derived from DHA and EPA is not possible while the diet for feeding studies can be modulated accordingly.

Given the large amount of biologically relevant effects observed in studies using *fat-1* mice, these results indicate that efficacy of n3-PUFA, and their derived oxylipins, might thus be found already in the context of rather low endogenous levels of n3-PUFA which could be easily achieved—and even surpassed—by dietary interventions. However, for some questions, e.g. the in depth and concentration dependent effects of (individual) n3-PUFA *in vivo*, feeding studies remain the model of choice.

## Supporting information

S1 Fig Bodyweight of animals during the feeding period. (TIF)

S2 Fig Relative distribution of fatty acid classes in tissues. (TIF)

S3 Fig Mean %difference of relative levels of EPA (A), DHA (B), n3 DPA (C) and ARA (D) in WT animals after 30 days on a sunflower oil based diet enriched with EPA and DHA in comparison to baseline (WT mice, D0). (TIF)

S4 Fig Mean% difference of relative levels of EPA (A), DHA (B), n3 DPA (C) and ARA (D) in WT animals after 30 days on a sunflower oil based diet enriched with EPA and DHA in comparison to *fat-1* mice after 30 days on standard sunflower diet. (TIF)

S5 Fig %EPA+DHA in blood fractions (A) and in tissues (B). (TIF)

S6 Fig Mean% difference of selected oxylipin concentrations in (A) plasma (B) brain and (C) colon in WT animals after 30 days on a sunflower oil based diet enriched with EPA and DHA in comparison to baseline (WT mice, D0). (TIF)

S7 Fig Sum (I) and relative profile (II) of all EPA, DHA and ARA derived oxylipins covered by the LC-MS method. (TIF)

S8 Fig Mean %difference of selected oxylipin concentrations in (A) plasma (B) brain and (C) colon in WT animals after 30 days on a sunflower oil based diet enriched with EPA and DHA (WT-STD+n3) in comparison to *fat-1* mice after 30 days on standard sunflower diet. (TIF)

S1 Table Determined fat content, peroxide value and fatty acid composition of the experimental diets. (PDF)

S2 Table Fatty acid composition of the standard mouse diet. (PDF)

S3 Table Absolute concentrations of fatty acids in blood (A) and relative distribution of fatty acids in blood and tissues (B) of the experimental groups. (XLSX)

S4 Table n6/n3 ratio and %EPA+DHA in blood and tissues of the experimental groups. (XLSX)

S5 Table Absolute concentrations of oxylipins in plasma, colon and brain of the experimental groups. (XLSX)

S6 Table Statistical comparison of the fatty acid profile during the course of the feeding experiment with the n3-PUFA enriched diet vs D0. (PDF)

S7 Table Statistical comparison of the fatty acid profile in transgenic fat-1 mice and wild type animals (WT-STD) on a standard sunflower oil based diet, as well as in wild type mice on the same diet enriched with EPA and DHA (WT-STD+n3) after 30 days of feeding. (PDF)

S8 Table Statistical comparison of the oxylipin profile in fat-1 mice and wild type animals (WT-STD) on a standard sunflower oil based diet, as well as in wild type mice on the same diet enriched with EPA and DHA (WT-STD+n3) after 30 days of feeding. (PDF)

## Abbreviations

ALA: alpha linolenic acid
ARA: arachidonic acid
COX: cyclooxygenase
CYP: cytochrom P450
DHA: docosahexaenoic acid
DiHDPE: dihydroxy docosapentaenoic acid
DiH-FA: dihydroxy fatty acid
EPA: eicosapentaenoic acid
EpDPE: epoxy docosapentaenoic acid
EpETE: epoxy eicosatetraenoic acid
EpETrE: epoxy eicosatrienoic acid
Ep-FA: epoxy fatty acid
GC-FID: gas chromatography flame ionization detection
HDHA: hydroxy docosahexaenoic acid
HEPE: hydroxy eicosapentaenoic acid
HETE: hydroxy eicosatetraenoic acid
LA: linoleic acid
LC-MS: liquid chromatography mass spectrometry
LOQ: limit of quantification
LOX: lipoxygenase
LT: leukotriene
MUFA: monounsaturated fatty acid
n3-DPA: n3 docosapentaenoic acid
OH-FA: hydroxy fatty acid
PG: prostaglandin
PUFA: polyunsaturated fatty acid
Rv: resolvin
SEM: standard error of the mean
SFA: saturated fatty acid
SPM: specialized pro-resolving lipid mediator
Tx: thromboxane
WT: wild type
