# Supplementary figures and images for: Modulation of the endogenous omega-3 fatty acid and oxylipin profile in vivo—A comparison of the fat-1 transgenic mouse with C57BL/6 wildtype mice on an omega-3 fatty acid enriched diet
Source: PLoS One. 2017 Sep 8;12(9):e0184470. doi: 10.1371/journal.pone.0184470 (PMC5590967; doi:10.1371/journal.pone.0184470)

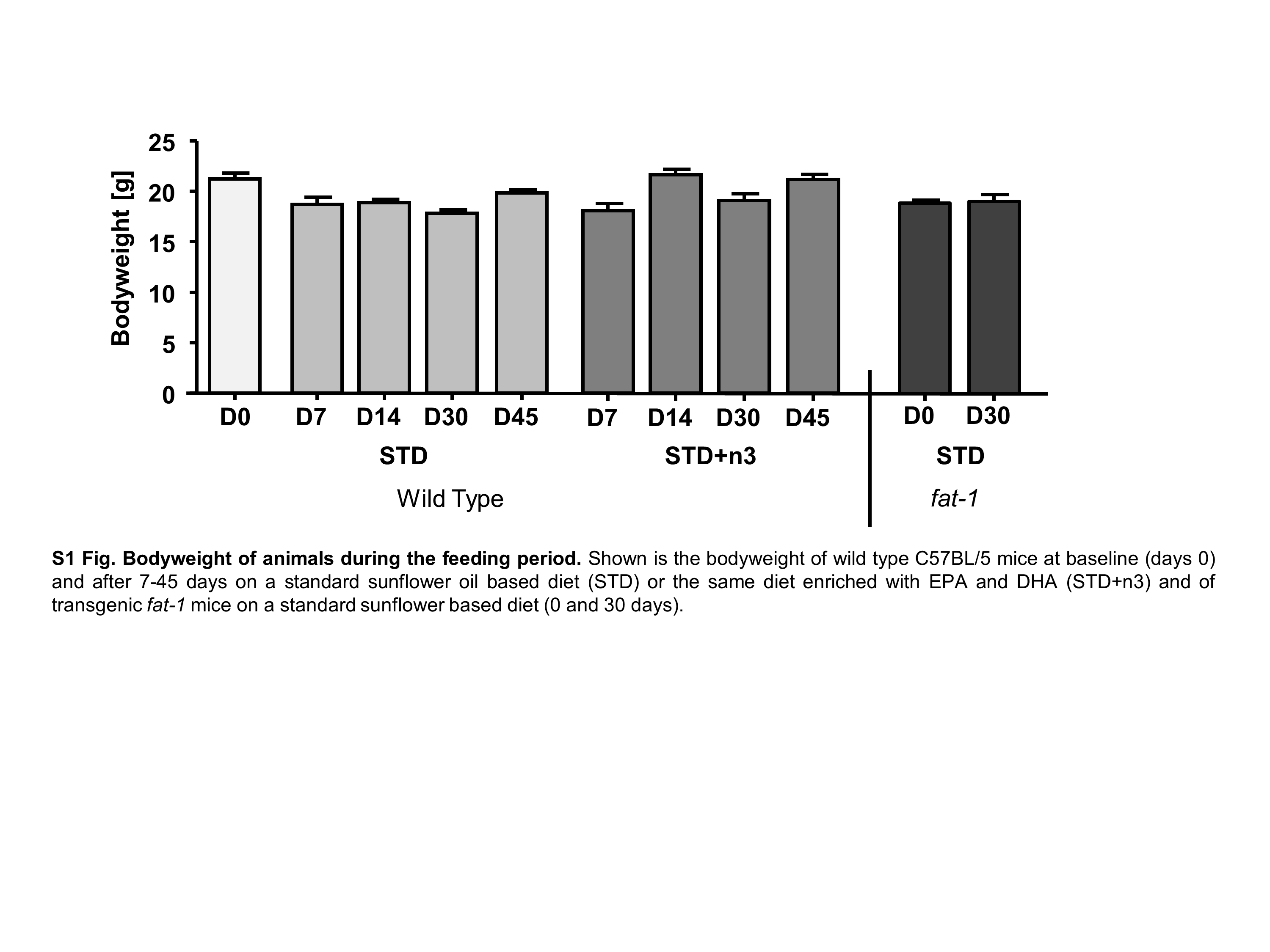

Supplement: S1 Fig — (TIF) [file pone.0184470.s001.tif]

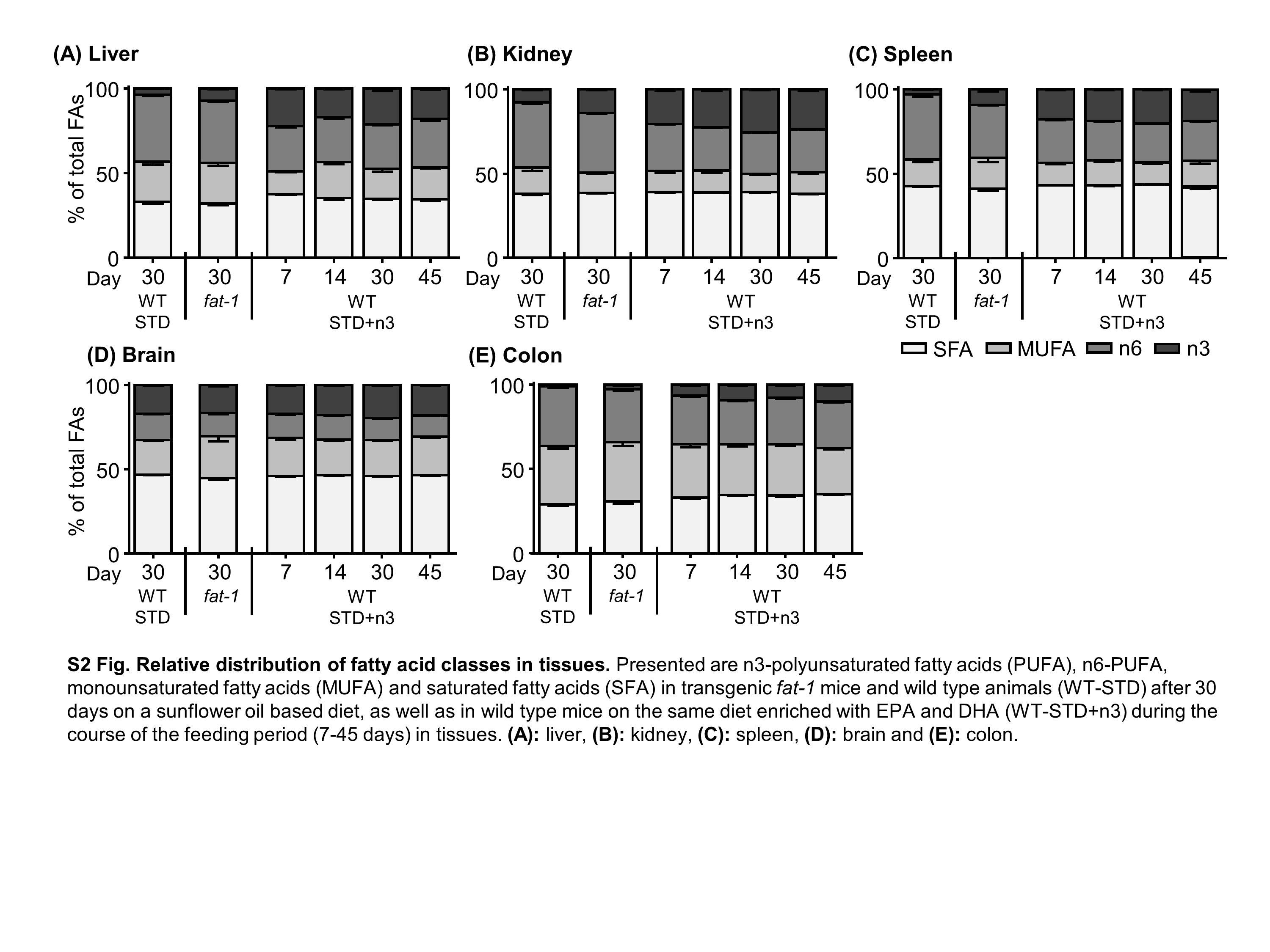

Supplement: S2 Fig — (TIF) [file pone.0184470.s002.tif]

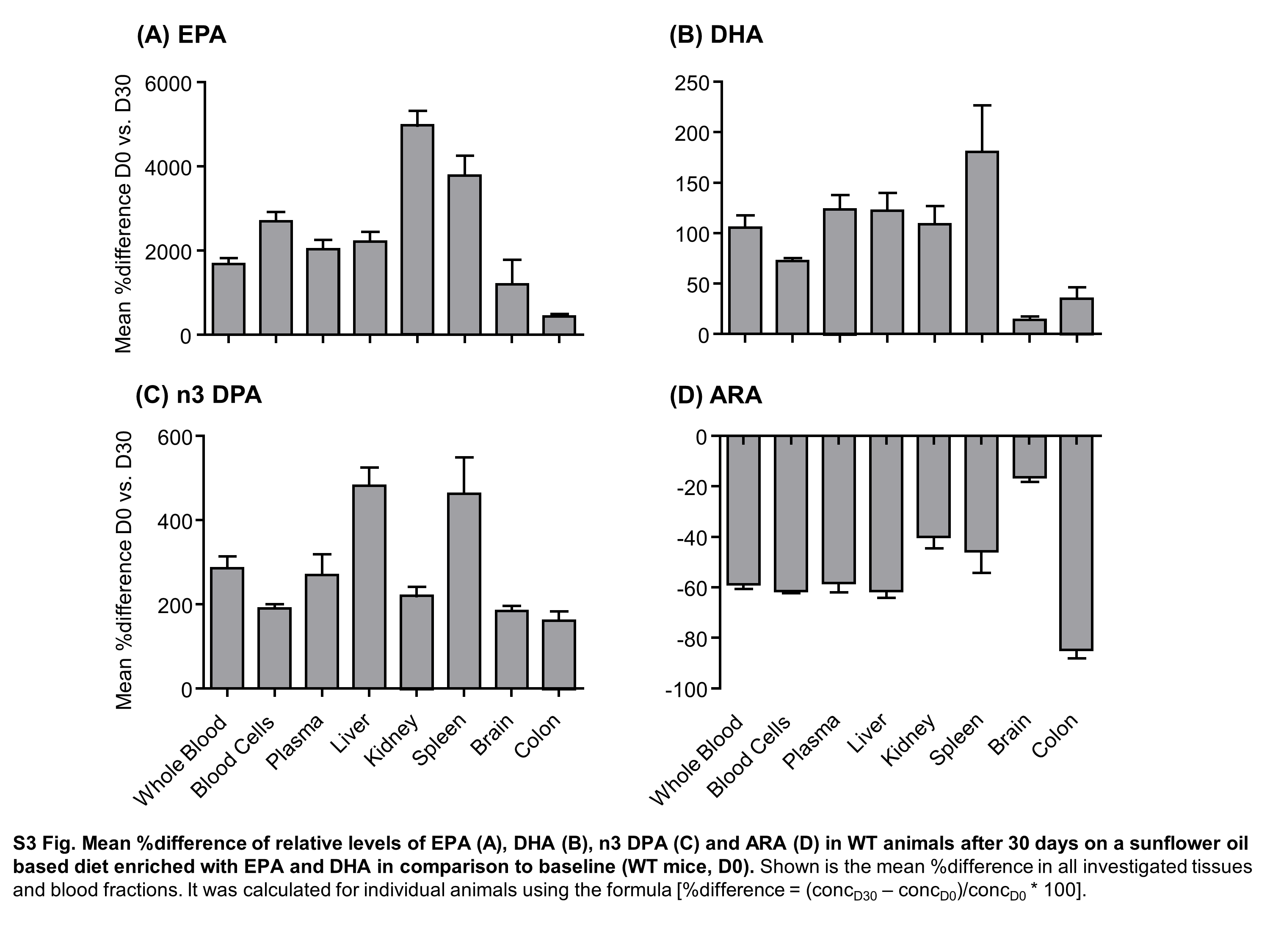

Supplement: S3 Fig — (TIF) [file pone.0184470.s003.tif]

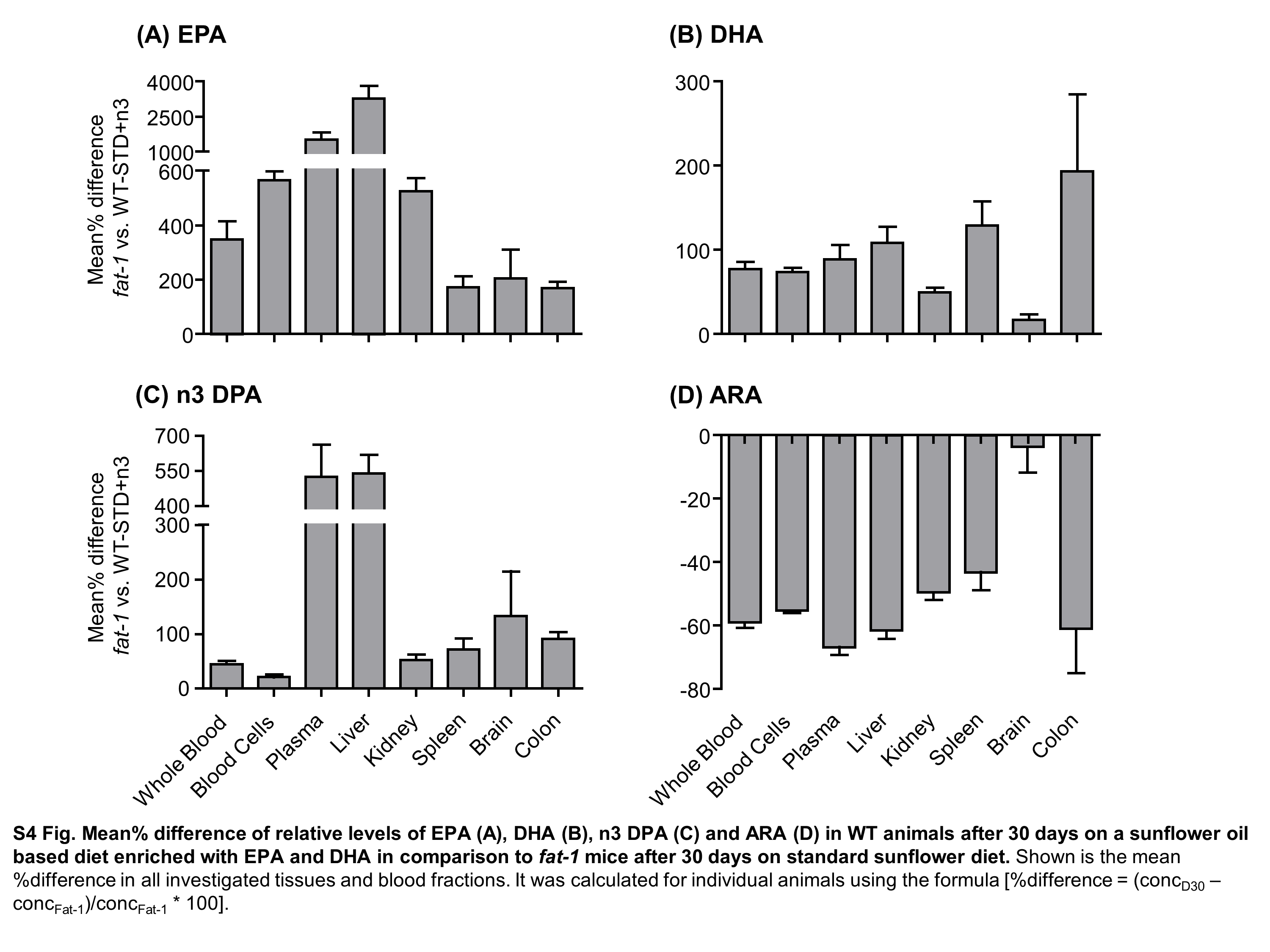

Supplement: S4 Fig — (TIF) [file pone.0184470.s004.tif]

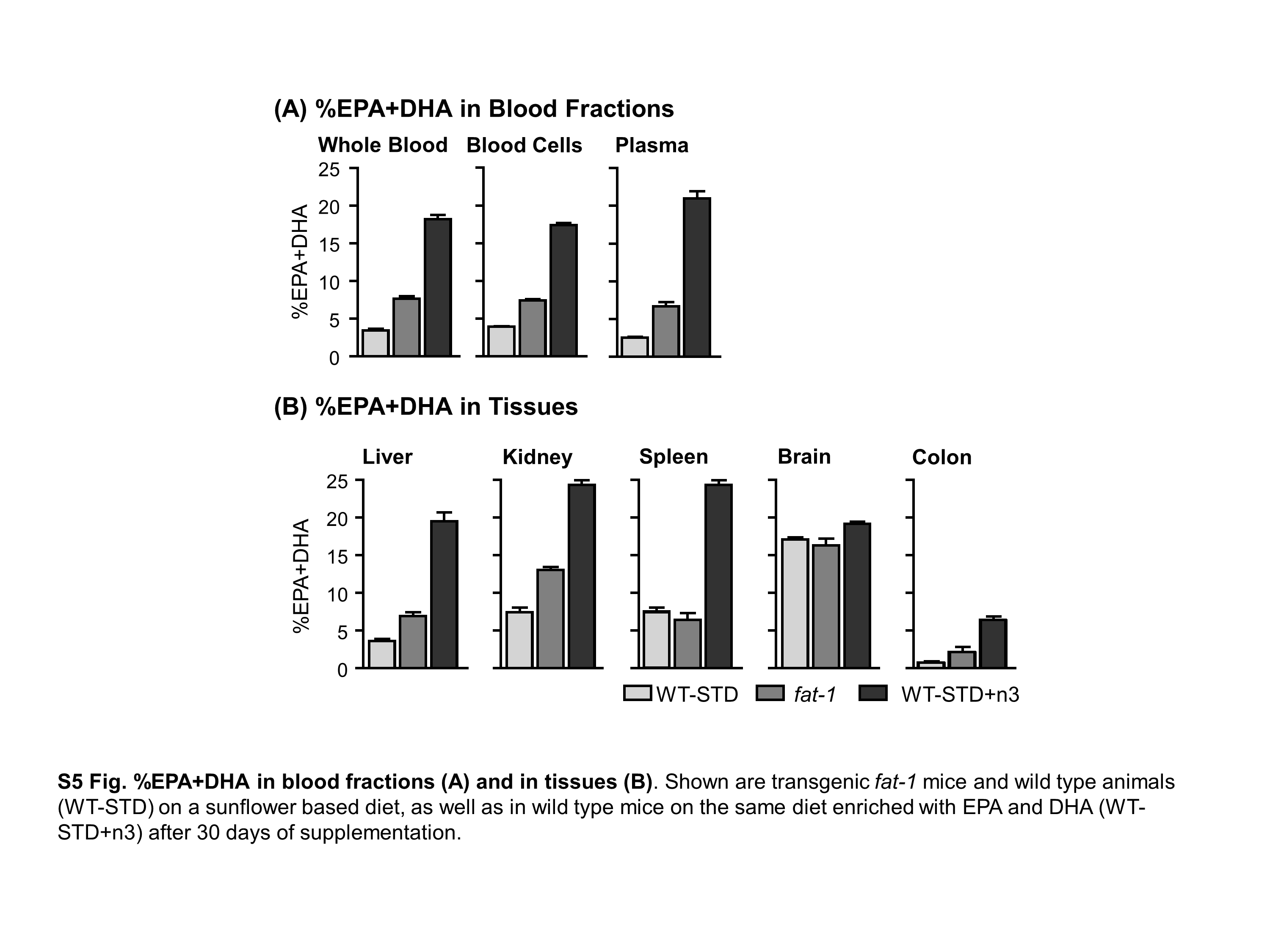

Supplement: S5 Fig — (TIF) [file pone.0184470.s005.tif]

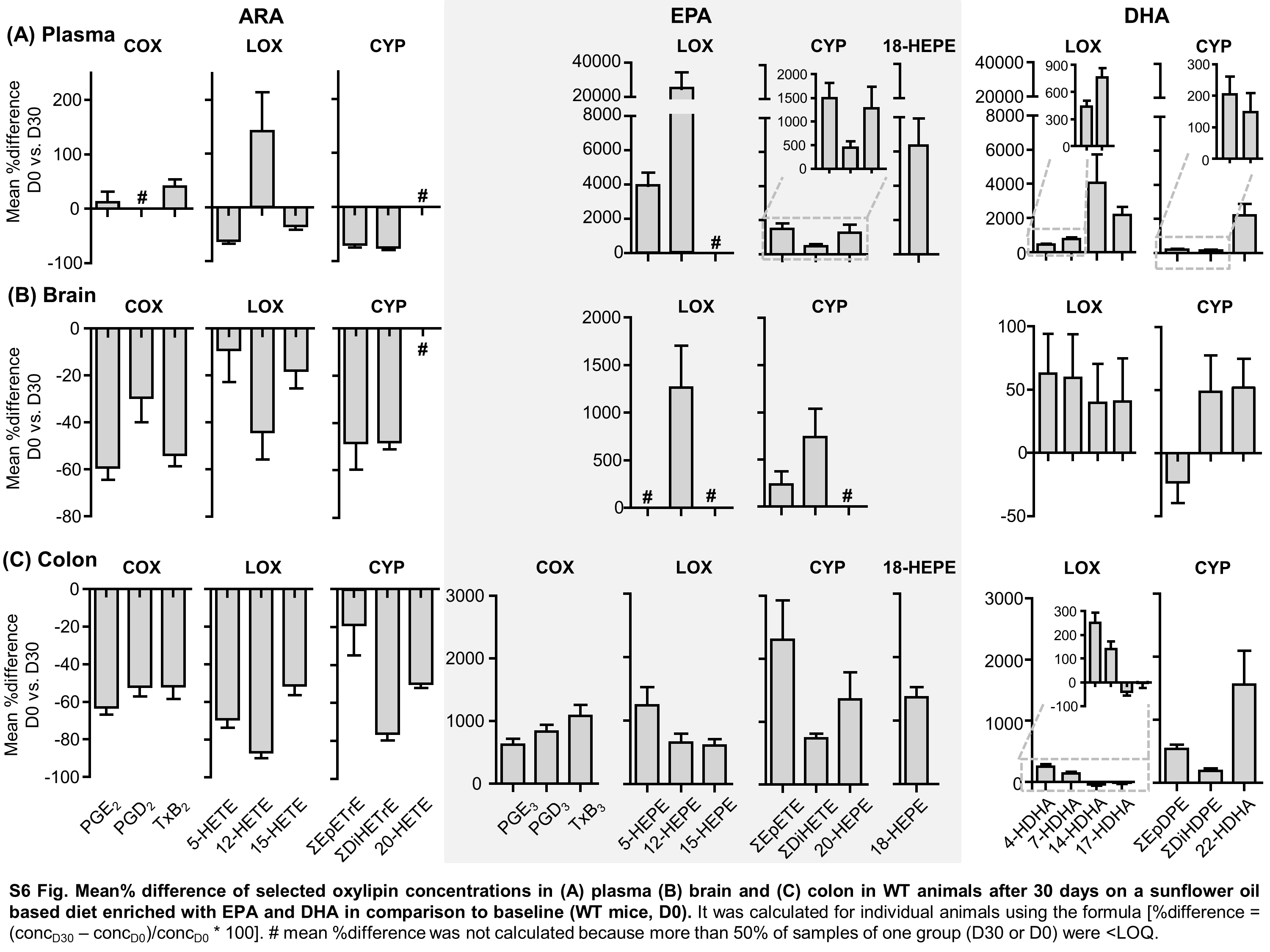

Supplement: S6 Fig — (TIF) [file pone.0184470.s006.tif]

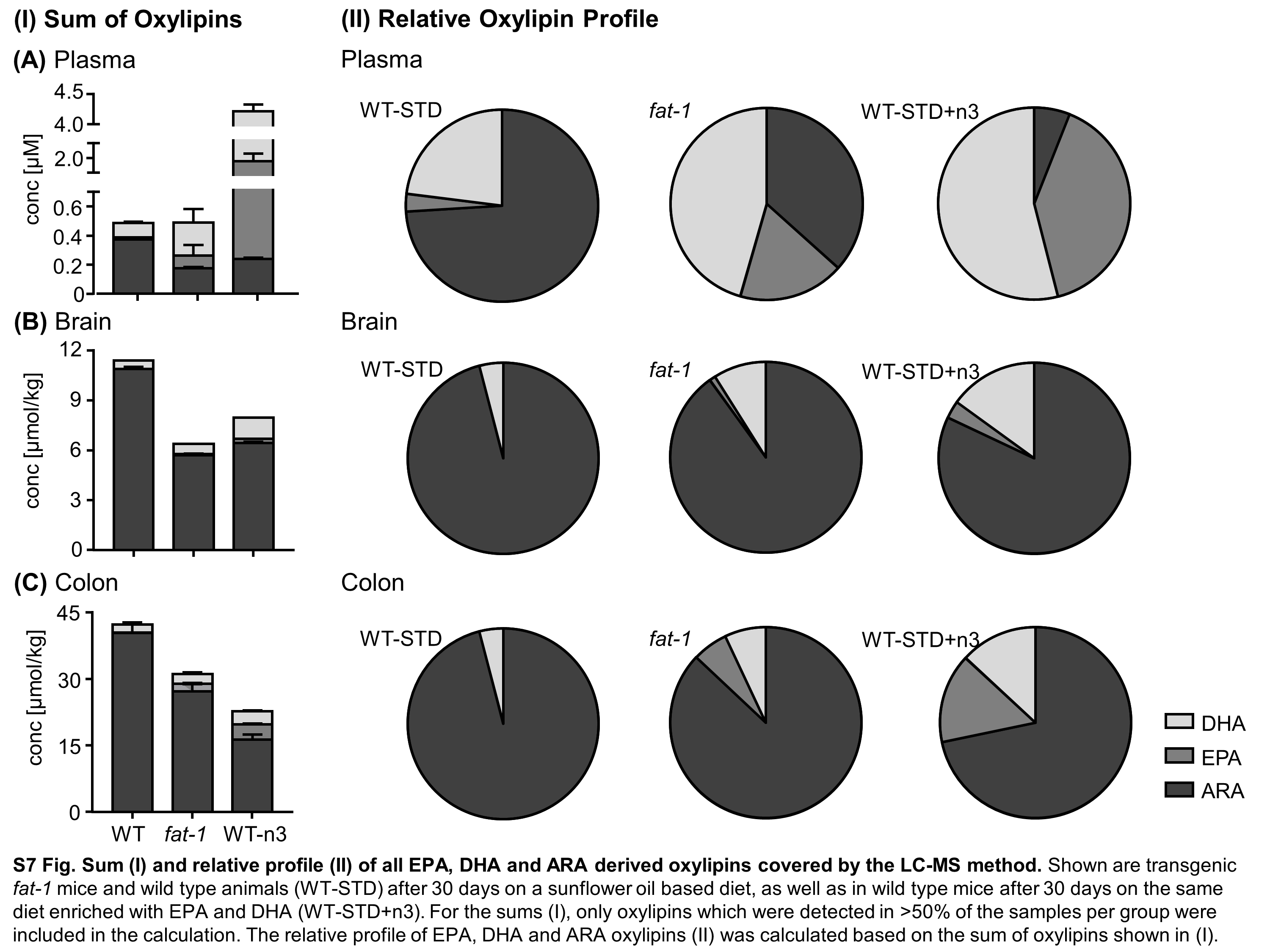

Supplement: S7 Fig — (TIF) [file pone.0184470.s007.tif]

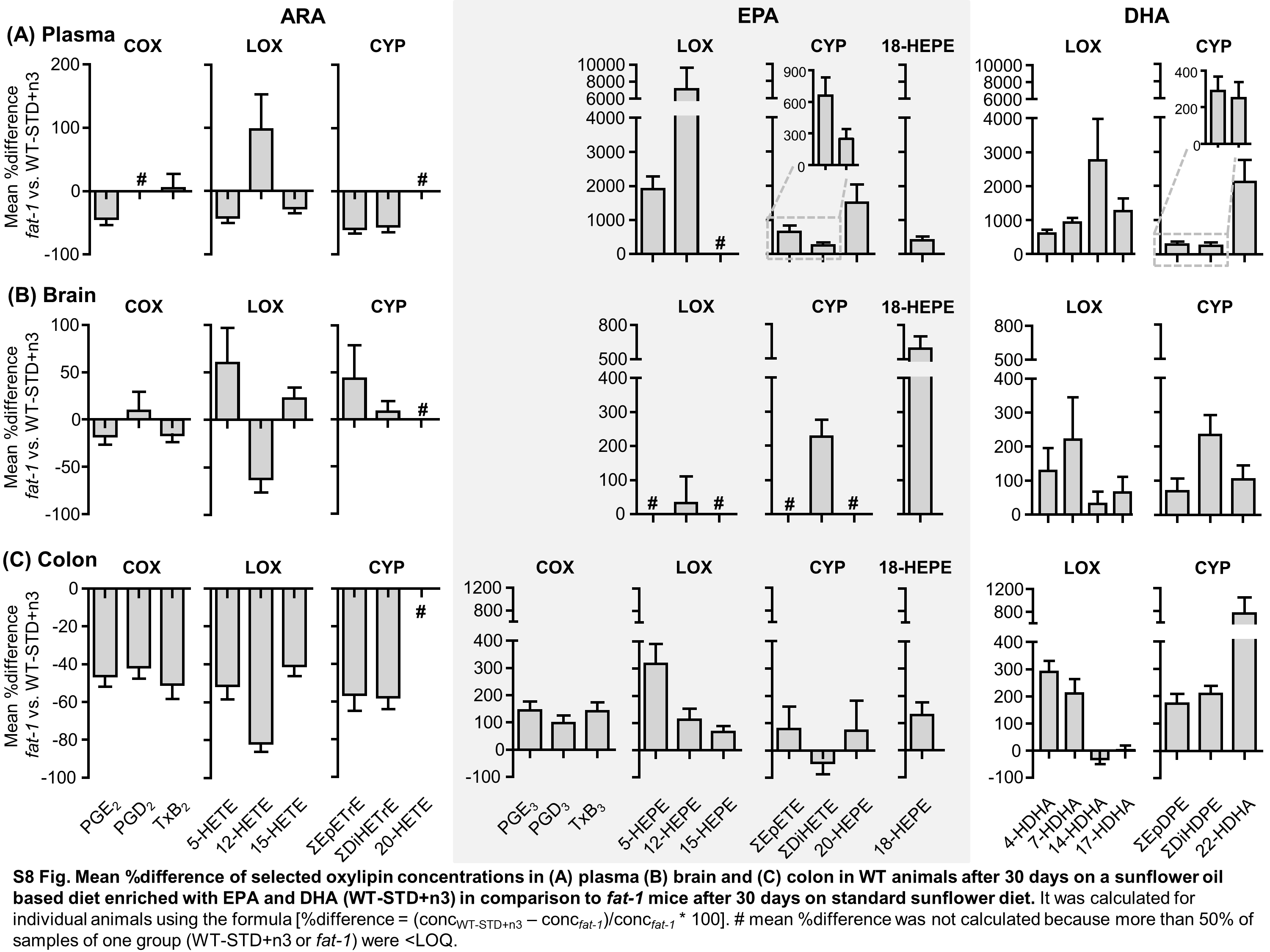

Supplement: S8 Fig — (TIF) [file pone.0184470.s008.tif]
