# Supplementary material for: Modulation of the endogenous omega-3 fatty acid and oxylipin profile in vivo—A comparison of the fat-1 transgenic mouse with C57BL/6 wildtype mice on an omega-3 fatty acid enriched diet
Source: PLoS One. 2017 Sep 8;12(9):e0184470. doi: 10.1371/journal.pone.0184470 (PMC5590967; doi:10.1371/journal.pone.0184470)
Supplement: S1 Table — (PDF) [file pone.0184470.s009.pdf]

S1 Table. Determined fat content#, peroxide value+ and fatty acid composition* of the experimental diets.

Diets were based on a standard experimental diet from ssniff Spezialdiäten GmbH (product number: E15051; with 17.6% crude protein, 5.0% crude fiber, 5.3% crude ash, 31.4% starch, 11.0% sugar and 10.1% fat as specified by the manufacturer). All results are shown as mean ± deviation from the mean (n=2).

	STD	STD+n3
Determined **fat content** in the diet [g/100g]	9.7 ± 0.2	9.9 ± 0.2
**Peroxide value** [mEq O2/kg]	2.6 ± 0.1	3.6 ± 0.1
**[%] Fatty Acid** in lipid extract		
C14:0	0.15 ± 0.05	0.19 ± 0.04
C15:0	0.07 ± 0.02	-
C16:0	7.995 ± 0.008	6.61 ± 0.05
C16:1n7	0.217 ± 0.008	0.24 ± 0.03
C17:0	0.071 ± 0.007	0.058 ± 0.007
C18:0	3.288 ± 0.001	2.64 ± 0.03
C18:1n9	27.064 ± 0.004	21.65 ± 0.06
C18:1n7	1.26 ± 0.04	1.047 ± 0.003
C18:2n6	58.306 ± 0.003	46.5 ± 0.1
C18:3n3	0.335 ± 0.005	0.287 ± 0.003
C20:0	0.218 ± 0.003	0.184 ± 0.004
C20:1n9	0.167 ± 0.002	0.14 ± 0.01
C20:4n6	-	0.303 ± 0.003
C20:5n3	-	8.91 ± 0.07
C22:0	0.653 ± 0.008	0.95 ± 0.03
C22:5n3	-	1.18 ± 0.05
C24:0	0.204 ± 0.008	-
C22:6n3	-	8.91 ± 0.09
C24:1n9	-	0.17 ± 0.02

#For the determination of the fatty acid composition and the peroxide value, lipids were first extracted from the pellets according to the Weibull-Stoldt method, performed as rapid microextraction [1].

+Determination of the peroxide value was performed by iodometry as described [3]. Briefly, after dissolving the lipid extract in acetic acid/isooctane (3:2, *v:v*) and addition of potassium iodide the formed iodine titrated with sodium thiosulfate.

*The fatty acid composition was determined after derivatization to fatty acid methyl esters by means of gas chromatography with flame ionization detection using response factors [2].

References

1. Schulte E. Vereinfachte Mikromethode zur gravimetrischen Bestimmung des Fettgehaltes von Lebensmittels nach Säureaufschluss (Kurzmitteilung). Deutsche Lebensmittelrundschau. 2004;100(5):188-9.

2. Ostermann AI, Muller M, Willenberg I, Schebb NH. Determining the fatty acid composition in plasma and tissues as fatty acid methyl esters using gas chromatography - a comparison of different derivatization and extraction procedures. Prostaglandins Leukot Essent Fatty Acids. 2014;91(6):235-41.

3. Untersuchung von Lebensmitteln, Bestimmung der Peroxidzahl in tierischen und pflanzlichen Fetten und Ölen, Iodometrische (visuelle) Endpunktbestimmung (13.00-37). In Amtliche Sammlung von Untersuchungsverfahren nach § 64 LFBG, 2012; BVL.
