# Supplementary material for: Modulation of the endogenous omega-3 fatty acid and oxylipin profile in vivo—A comparison of the fat-1 transgenic mouse with C57BL/6 wildtype mice on an omega-3 fatty acid enriched diet
Source: PLoS One. 2017 Sep 8;12(9):e0184470. doi: 10.1371/journal.pone.0184470 (PMC5590967; doi:10.1371/journal.pone.0184470)
Supplement: S2 Table — (PDF) [file pone.0184470.s010.pdf]

S2 Table. Fatty acid composition of the standard mouse diet.

Shown are specifications according to the manufacturer ssniff Spezialdiäten GmbH (product number: V1530; with 19.0% crude protein, 4.9% crude fiber, 6.4% crude ash, 36.5% starch, 4.7% sugar and 3.3% fat as specified by the manufacturer).

	**[%] Fatty Acid** in diet
C14:0	0.01
C16:0	0.47
C16:1	0.01
C18:0	0.08
C18:1	0.62
C18:2	1.80
C18:3	0.23
C20:0	0.01
C20:1	0.02
C20:5	-
C22:6	-
