# Supplementary material for: Modulation of the endogenous omega-3 fatty acid and oxylipin profile in vivo—A comparison of the fat-1 transgenic mouse with C57BL/6 wildtype mice on an omega-3 fatty acid enriched diet
Source: PLoS One. 2017 Sep 8;12(9):e0184470. doi: 10.1371/journal.pone.0184470 (PMC5590967; doi:10.1371/journal.pone.0184470)
Supplement: S6 Table — (PDF) [file pone.0184470.s014.pdf]

S6 Table: Statistical comparison of the fatty acid profile during the course of the feeding experiment with the n3-PUFA enriched diet vs D0. Statistical differences were determined using one-way ANOVA followed by Dunnett's post test (* p<0.05, ** p<0.01, *** p<0.001, **** p<0.0001; n.s. = not significant).

		EPA	DHA	ARA	n6-PUFA	n3-PUFA	MUFA	SFA
Whole Blood	D7	****	****	****	****	****	*	n.s.
	D14	****	****	****	****	****	**	n.s.
	D30	****	****	****	****	****	**	n.s.
	D45	****	****	****	****	****	***	n.s.
Plasma	D7	****	****	****	****	****	****	n.s.
	D14	****	****	****	****	****	****	n.s.
	D30	****	****	****	****	****	****	**
	D45	****	****	****	****	****	****	n.s.
Blood Cells	D7	****	****	****	****	****	***	*
	D14	****	****	****	****	****	n.s.	**
	D30	****	****	****	****	****	**	**
	D45	****	****	****	****	****	*	***
Liver	D7	****	****	****	****	****	****	***
	D14	****	****	****	****	****	*	n.s.
	D30	****	****	****	****	****	***	n.s.
	D45	****	****	****	**	****	**	n.s.
Kidney	D7	****	****	**	****	****	***	n.s.
	D14	****	****	****	****	****	**	n.s.
	D30	****	****	****	****	****	****	n.s.
	D45	****	****	****	****	****	***	n.s.
Spleen	D7	****	****	n.s.	n.s.	****	***	*
	D14	****	****	**	**	****	**	*
	D30	****	****	***	***	****	***	**
	D45	****	****	****	***	****	**	n.s.
Brain	D7	****	n.s.	*	n.s.	n.s.	n.s.	n.s.
	D14	****	n.s.	n.s.	n.s.	n.s.	n.s.	n.s.
	D30	****	**	****	****	***	n.s.	n.s.
	D45	****	n.s.	****	****	n.s.	n.s.	n.s.
Colon	D7	**	n.s.	****	n.s.	*	n.s.	****
	D14	****	**	****	***	****	n.s.	**
	D30	**	n.s.	****	*	**	n.s.	**
	D45	****	****	****	*	****	n.s.	*
