# Supplementary material for: Modulation of the endogenous omega-3 fatty acid and oxylipin profile in vivo—A comparison of the fat-1 transgenic mouse with C57BL/6 wildtype mice on an omega-3 fatty acid enriched diet
Source: PLoS One. 2017 Sep 8;12(9):e0184470. doi: 10.1371/journal.pone.0184470 (PMC5590967; doi:10.1371/journal.pone.0184470)
Supplement: S7 Table — (PDF) [file pone.0184470.s015.pdf]

S7 Table: Statistical comparison of the fatty acid profile in transgenic *fat-1* mice and wild type animals (WT-STD) on a standard sunflower oil based diet, as well as in wild type mice on the same diet enriched with EPA and DHA (WT-STD+n3) after 30 days of feeding. Statistical differences were determined using one-way ANOVA followed by Tukey's post test (* p<0.05, ** p<0.01, *** p<0.001, **** p<0.0001; n.s. = not significant).

		EPA	DHA	ARA	n6-PUFA	n3-PUFA	MUFA	SFA
Whole Blood	WT-STD vs *fat-1*	ns	****	****	****	****	n.s.	n.s.
	WT-STD vs STD+n3	****	****	****	****	****	n.s.	***
	fat-1 vs WT-STD+n3	****	****	****	****	****	n.s.	**
Plasma	WT-STD vs *fat-1*	+	****	*	***	**	n.s.	n.s.
	WT-STD vs STD+n3	+	****	****	****	****	n.s.	n.s.
	fat-1 vs WT-STD+n3	****	****	****	****	****	n.s.	n.s.
Blood Cells	WT-STD vs *fat-1*	+	****	****	****	****	n.s.	n.s.
	WT-STD vs STD+n3	+	****	****	****	****	n.s.	**
	fat-1 vs WT-STD+n3	****	****	****	****	****	n.s.	*
Liver	WT-STD vs *fat-1*	+	**	*	*	*	n.s.	n.s.
	WT-STD vs STD+n3	+	****	****	****	****	n.s.	n.s.
	fat-1 vs WT-STD+n3	****	****	****	****	****	*	n.s.
Kidney	WT-STD vs *fat-1*	**	****	n.s.	**	****	n.s.	n.s.
	WT-STD vs STD+n3	****	****	****	****	****	*	n.s.
	fat-1 vs WT-STD+n3	****	****	****	****	****	n.s.	n.s.
Spleen	WT-STD vs *fat-1*	****	**	***	****	****	n.s.	n.s.
	WT-STD vs STD+n3	****	****	****	****	****	n.s.	n.s.
	fat-1 vs WT-STD+n3	****	****	***	****	****	n.s.	n.s.
Brain	WT-STD vs *fat-1*	+	n.s.	n.s.	n.s.	n.s.	n.s.	n.s.
	WT-STD vs STD+n3	+	n.s.	*	*	*	n.s.	n.s.
	fat-1 vs WT-STD+n3	****	**	n.s.	n.s.	**	n.s.	n.s.
Colon	WT-STD vs *fat-1*	n.s.	n.s.	n.s.	**	n.s.	n.s.	n.s.
	WT-STD vs STD+n3	****	****	*	****	****	n.s.	**
	fat-1 vs WT-STD+n3	****	****	n.s.	**	****	n.s.	*

+ For analytes that were not detectable in the WT-STD group, a t-test (two-tailed, unpaired), with Welch correction in case of unequal variances, was performed comparing *fat-1* and WT-STD+n3 groups.
