# Supplementary material for: Modulation of the endogenous omega-3 fatty acid and oxylipin profile in vivo—A comparison of the fat-1 transgenic mouse with C57BL/6 wildtype mice on an omega-3 fatty acid enriched diet
Source: PLoS One. 2017 Sep 8;12(9):e0184470. doi: 10.1371/journal.pone.0184470 (PMC5590967; doi:10.1371/journal.pone.0184470)
Supplement: S8 Table — (PDF) [file pone.0184470.s016.pdf]

S8 Table: Statistical comparison of the oxylipin profile in *fat-1* mice and wild type animals (WT-STD) on a standard sunflower oil based diet, as well as in wild type mice on the same diet enriched with EPA and DHA (WT-STD+n3) after 30 days of feeding. Statistical analysis was conducted using one-way ANOVA followed by Tukey's post test (* p<0.05, ** p<0.01, *** p<0.001, **** p<0.0001; n.s. = not significant).

A) Oxylipin Profile in Plasma

			WT-STD vs *fat-1*	STD vs WT-STD+n3	*fat-1* vs WT-STD+n3
**ARA**	**Prostanoids**	6-keto-$PGF_{1\alpha}$	n.s.	n.s.	n.s.
		$PGF_{2\alpha}$[a]	-	-	-
		PGE_2	n.s.	**	n.s.
		PGD_2[b]	-	-	-
		PGJ_2[a]	-	-	-
		PGB_2[a]	-	-	-
		15-deoxy-PGJ_2[a]	-	-	-
		11b-$PGF_{2\alpha}$[a]	-	-	-
		13,14dih-15k-$PGF_{2\alpha}$	n.s.	*	n.s.
		TxB_2	n.s.	n.s.	n.s.
	**Epoxy-FA**	5(6)-EpETrE	n.s.	**	*
		8(9)-EpETrE	n.s.	*	*
		11(12)-EpETrE	n.s.	****	***
		14(15)-EpETrE	n.s.	****	***
	**Dihydroxy-FA**	5,6-DiHETrE	n.s.	**	n.s.
		8,9-DiHETrE	n.s.	**	*
		11,12-DiHETrE	n.s.	*	n.s.
		14,15-DiHETrE	n.s.	n.s.	n.s.
	**Hydroxy-FA**	5-HETE	n.s.	*	n.s.
		8-HETE	n.s.	*	**
		9-HETE[a]	-	-	-
		11-HETE	*	**	n.s.
		12-HETE	n.s.	n.s.	n.s.
		15-HETE	n.s.	**	n.s.
		20-HETE[b]	-	-	-
	**Multiple Hydroxylated FA**	LTB_4[a]	-	-	-
		6-trans-LTB_4[a]	-	-	-
		20-COOH-LTB_4[a]	-	-	-
		20-OH-LTB_4[a]	-	-	-
		LXA_4[a]	-	-	-
		5,15-DiHETE[a]	-	-	-
		8,15-DiHETE[a]	-	-	-
	**Isoprostanes**	5-$iPF_{2\alpha}$[b]	-	-	-
		8-i-$PGF_{2\alpha}$[a]	-	-	-
	**Others**	5-oxo-ETE[a]	-	-	-
		15-oxo-ETE[d]	n.s.	-	-

		11,12-,15-TriHETrE[a]	-	-	-
		THF diol[a]	-	-	-
**EPA**	**Prostanoids**	PGE_3[a]	-	-	-
		PGD_3[a]	-	-	-
		d17-6k-$PGF_{1\alpha}$[a]	-	-	-
		TxB_3[c]	-	-	-
	**Epoxy-FA**	8(9)-EpETE[c]	-	-	-
		11(12)-EpETE[c]	-	-	-
		14(15)-EpETE[d]	-	-	**
		17(18)-EpETE[d]	-	-	*
	**Dihydroxy-FA**	5,6-DiHETE[a]	-	-	-
		8,9-DiHETE[a]	-	-	-
		11,12-DiHETE[d]	-	-	*
		14,15-DiHETE	n.s.	***	***
		17,18-DiHETE	n.s.	***	**
	**Hydroxy-FA**	5-HEPE[d]	-	-	**
		8-HEPE[d]	-	-	*
		12-HEPE	n.s.	**	**
		15-HEPE[c]	-	-	-
		18-HEPE[d]	-	-	**
		19-HEPE	n.s.	****	***
		20-HEPE	n.s.	**	**
	**Multiple Hydroxylated FA**	LTB_5[a]	-	-	-
		RvE1[a]	-	-	-
		RvE2[a]	-	-	-
		18(S)-RvE3[a]	-	-	-
		18(R)-RvE3[a]	-	-	-
	**Others**	12-OH-17(18)-EpETE[a]	-	-	-
**DHA**	**Epoxy-FA**	10(11)-EpDPE	n.s.	***	**
		13(14)-EpDPE	n.s.	****	***
		16(17)-EpDPE	n.s.	****	***
		19(20)-EpDPE	n.s.	****	***
	**Dihydroxy-FA**	4,5-DiHDPE	n.s.	****	***
		7,8-DiHDPE	n.s.	****	***
		10,11-DiHDPE	n.s.	****	***
		13,14-DiHDPE	n.s.	****	***
		16,17-DiHDPE	n.s.	***	**
		19,20-DiHDPE	n.s.	**	**
	**Hydroxy-FA**	4-HDHA	n.s.	****	****
		7-HDHA	n.s.	****	****
		8-HDHA	n.s.	****	****
		10-HDHA	n.s.	****	***
		11-HDHA	n.s.	****	****
		13-HDHA	n.s.	****	****
		14-HDHA	n.s.	**	**
		16-HDHA	n.s.	****	****

		17-HDHA	n.s.	****	****
		20-HDHA	n.s.	****	****
		21-HDHA	n.s.	***	***
		22-HDHA	n.s.	**	**
	**Multiple Hy-droxylated FA**	RvD1[a]	-	-	-
**LA**	**Epoxy-FA**	9(10)-EpOME	n.s.	n.s.	n.s.
		12(13)-EpOME	n.s.	n.s.	n.s.
	**Dihydroxy-FA**	9,10-DiHOME	n.s.	n.s.	n.s.
		12,13-DiHOME	n.s.	n.s.	n.s.
	**Hydroxy-FA**	9-HODE	n.s.	n.s.	n.s.
		13-HODE	*	n.s.	n.s.
	**Others**	EKODE	n.s.	n.s.	n.s.
		9-oxo-ODE	n.s.	n.s.	n.s.
		13-oxo-ODE	n.s.	n.s.	n.s.
		9,12,13-TriHOME	n.s.	n.s.	n.s.
		9,10,13-TriHOME	n.s.	n.s.	n.s.
**ALA**	**Epoxy-FA**	9(10)-EpODE	n.s.	n.s.	n.s.
		12(13)-EpODE[d]	-	-	n.s.
		15(16)-EpODE	n.s.	n.s.	n.s.
	**Dihydroxy-FA**	9,10-DiHODE	*	n.s.	n.s.
		12,13-DiHODE[a]	-	-	-
		15,16-DiHODE	n.s.	n.s.	n.s.
	**Hydroxy-FA**	9-HOTrE	n.s.	n.s.	n.s.
		13-HOTrE	n.s.	n.s.	n.s.
**DGLA**	**Prostanoids**	PGE_1[a]	-	-	-
		PGD_1[a]	-	-	-
		$PGF_{1\alpha}$[a]	-	-	-
		15-k-$PGF_{1\alpha}$[a]	-	-	-
		13,14-dih-15-k-PGE_1[a]	-	-	-
		TxB_1[a]	-	-	-
	**Hydroxy-FA**	15(S)-HETrE	n.s.	n.s.	n.s.
**Oleic Acid**		9(10)-ep-stearic acid	n.s.	n.s.	n.s.
		9,10-dih-stearic acid	n.s.	n.s.	n.s.
**C20:3 n9**		LTB_3[a]	-	-	-
**C22:4 n6**		dihomo-$PGF_{2\alpha}$[a]	-	-	-

[a] analyte below LOQ in all groups; [b] analyte below LOQ in WT-STD+n3 and *fat-1*; [c] analyte below LOQ in WT-STD and *fat-1*; [d] For analytes that were below LOQ one of the three groups, a t-test (two-tailed, unpaired), with Welch correction in case of unequal variances, was performed.

B) Oxylipin Profile in Brain

			WT-STD vs *fat-1*	STD vs WT-STD+n3	*fat-1* vs WT-STD+n3
**ARA**	**Prostanoids**	6-keto-PGF$_{1\alpha}$	*	***	n.s.
		PGF$_{2\alpha}$	***	****	n.s.
		PGE$_2$	****	****	n.s.
		PGD$_2$	****	****	n.s.
		PGJ$_2$	n.s.	n.s.	n.s.
		PGB$_2$[a]	-	-	-
		15-deoxy-PGJ$_2$[a]	-	-	-
		11b-PGF$_{2\alpha}$[a]	-	-	-
		13,14dih-15k-PGF$_{2\alpha}$	****	****	n.s.
		TxB$_2$	****	****	n.s.
	**Epoxy-FA**	5(6)-EpETrE	n.s.	n.s.	n.s.
		8(9)-EpETrE	n.s.	n.s.	n.s.
		11(12)-EpETrE	*	n.s.	n.s.
		14(15)-EpETrE	n.s.	n.s.	n.s.
	**Dihydroxy-FA**	5,6-DiHETrE[a]	-	-	-
		8,9-DiHETrE[a]	-	-	-
		11,12-DiHETrE	*	n.s.	n.s.
		14,15-DiHETrE	**	*	n.s.
	**Hydroxy-FA**	5-HETE	n.s.	n.s.	n.s.
		8-HETE	n.s.	n.s.	n.s.
		9-HETE	n.s.	n.s.	**
		11-HETE	****	****	n.s.
		12-HETE	n.s.	n.s.	n.s.
		15-HETE	****	***	n.s.
		20-HETE[b]	-	-	-
	**Multiple Hydroxylated FA**	LTB$_4$[a]	-	-	-
		6-trans-LTB$_4$[a]	-	-	-
		20-COOH-LTB$_4$[a]	-	-	-
		20-OH-LTB$_4$[a]	-	-	-
		LXA$_4$[a]	-	-	-
		5,15-DiHETE	***	n.s.	*
		8,15-DiHETE	***	n.s.	*
	**Isoprostanes**	5-iPF$_{2\alpha}$[a]	-	-	-
		8-i-PGF$_{2\alpha}$[a]	-	-	-
	**Others**	5-oxo-ETE[d]	n.s.	-	-
		15-oxo-ETE	n.s.	n.s.	n.s.
		11,12-,15-TriHETrE[b]	-	-	-
		THF diol[a]	-	-	-
**EPA**	**Prostanoids**	PGE$_3$[a]	-	-	-
		PGD$_3$[a]	-	-	-
		d17-6k-PGF$_{1\alpha}$[a]	-	-	-
		TxB$_3$[c]	-	-	-

	**Epoxy-FA**	8(9)-EpETE[a]	-	-	-
		11(12)-EpETE[c]	-	-	-
		14(15)-EpETE[c]	-	-	-
		17(18)-EpETE[a]	-	-	-
	**Dihydroxy-FA**	5,6-DiHETE[a]	-	-	-
		8,9-DiHET[a]	-	-	-
		11,12-DiHETE[a]	-	-	-
		14,15-DiHETE[a]	-	-	-
		17,18-DiHETE[d]	-	-	**
	**Hydroxy-FA**	5-HEPE[c]	-	-	-
		8-HEPE[c]	-	-	-
		12-HEPE	n.s.	n.s.	n.s.
		15-HEPE[c]	-	-	-
		18-HEPE[d]	-	-	****
		19-HEPE[c]	-	-	-
		20-HEPE[c]	-	-	-
	**Multiple Hydroxylated FA**	LTB_5[a]	-	-	-
		RvE1[a]	-	-	-
		RvE2[a]	-	-	-
		18(S)-RvE3[a]	-	-	-
		18(R)-RvE3[a]	-	-	-
	**Others**	12-OH-17(18)-EpETE[a]	-	-	-
**DHA**	**Epoxy-FA**	10(11)-EpDPE	n.s.	n.s.	n.s.
		13(14)-EpDPE	n.s.	n.s.	n.s.
		16(17)-EpDPE	n.s.	n.s.	n.s.
		19(20)-EpDPE	n.s.	n.s.	n.s.
	**Dihydroxy-FA**	4,5-DiHDPE[a]	-	-	-
		7,8-DiHDPE[a]	-	-	-
		10,11-DiHDPE[a]	-	-	-
		13,14-DiHDPE[a]	-	-	-
		16,17-DiHDPE[a]	-	-	-
		19,20-DiHDPE	n.s.	****	***
	**Hydroxy-FA**	4-HDHA	n.s.	**	*
		7-HDHA	n.s.	*	**
		8-HDHA	n.s.	***	**
		10-HDHA	n.s.	*	n.s.
		11-HDHA	n.s.	**	*
		13-HDHA	n.s.	n.s.	n.s.
		14-HDHA	n.s.	*	n.s.
		16-HDHA	n.s.	n.s.	n.s.
		17-HDHA	n.s.	n.s.	n.s.
		20-HDHA	n.s.	n.s.	n.s.
		21-HDHA	n.s.	****	**
		22-HDHA	n.s.	*	*
	**Multiple Hydroxylated FA**	RvD1[a]	-	-	-

**LA**	**Epoxy-FA**	9(10)-EpOME	n.s.	n.s.	n.s.
		12(13)-EpOME	n.s.	n.s.	n.s.
	**Dihydroxy-FA**	9,10-DiHOME	*	**	n.s.
		12,13-DiHOME	*	**	n.s.
	**Hydroxy-FA**	9-HODE	*	***	n.s.
		13-HODE	**	***	n.s.
	**Others**	EKODE	**	**	n.s.
		9-oxo-ODE	*	**	n.s.
		13-oxo-ODE[b]	-	-	-
		9,12,13-TriHOME	n.s.	*	n.s.
		9,10,13-TriHOME	n.s.	n.s.	n.s.
**ALA**	**Epoxy-FA**	9(10)-EpODE[b]	-	-	-
		12(13)-EpODE[a]	-	-	-
		15(16)-EpODE[b]	-	-	-
	**Dihydroxy-FA**	9,10-DiHODE[b]	-	-	-
		12,13-DiHODE[a]	-	-	-
		15,16-DiHODE[a]	-	-	-
	**Hydroxy-FA**	9-HOTrE	n.s.	*	n.s.
		13-HOTrE	*	*	n.s.
**DGLA**	**Prostanoids**	PGE_1[b]	-	-	-
		PGD_1	***	***	n.s.
		$PGF_{1\alpha}$	***	***	n.s.
		15-k-$PGF_{1\alpha}$[a]	-	-	-
		13,14-dih-15-k-PGE_1[a]	-	-	-
		TxB_1[a]	-	-	-
	**Hydroxy-FA**	15(S)-HETrE	n.s.	n.s.	n.s.
**Oleic Acid**		9(10)-ep-stearic acid	n.s.	n.s.	n.s.
		9,10-dih-stearic acid	n.s.	n.s.	n.s.
**C20:3 n9**		LTB_3[a]	-	-	-
**C22:4 n6**		dihomo-$PGF_{2\alpha}$[d]	**	-	-

[a] analyte below LOQ in all groups; [b] analyte below LOQ in WT-STD+n3 and *fat-1*; [c] analyte below LOQ in WT-STD and *fat-1*; [d] For analytes that were below LOQ one of the three groups, a t-test (two-tailed, unpaired), with Welch correction in case of unequal variances, was performed.

C) Oxylipin Profile in Colon

			WT-STD vs *fat-1*	STD vs WT-STD+n3	*fat-1* vs WT-STD+n3
**ARA**	**Prostanoids**	6-keto-$PGF_{1α}$	***	****	**
		$PGF_{2α}$	*	****	*
		PGE_2	****	****	****
		PGD_2	**	****	***
		PGJ_2	***	****	**
		PGB_2[a]	-	-	-
		15-deoxy-PGJ_2[d]	n.s.	-	-
		11b-$PGF_{2α}$[a]	-	-	-
		13,14dih-15k-$PGF_{2α}$[a]	-	-	-
		TxB_2	n.s.	****	***
	**Epoxy-FA**	5(6)-EpETrE	**	****	**
		8(9)-EpETrE	*	****	*
		11(12)-EpETrE	*	****	*
		14(15)-EpETrE	*	****	*
	**Dihydroxy-FA**	5,6-DiHETrE[d]	**	-	-
		8,9-DiHETrE	n.s.	***	***
		11,12-DiHETrE	n.s.	***	**
		14,15-DiHETrE	**	****	****
	**Hydroxy-FA**	5-HETE	****	****	**
		8-HETE	**	****	*
		9-HETE	**	****	**
		11-HETE	**	****	**
		12-HETE	**	****	n.s.
		15-HETE	**	****	*
		20-HETE[d]	n.s.	-	-
	**Multiple Hydroxylated FA**	LTB_4	***	****	n.s.
		6-trans-LTB_4	**	****	*
		20-COOH-LTB_4[d]	n.s.	-	-
		20-OH-LTB_4[a]	-	-	-
		LXA_4[a]	-	-	-
		5,15-DiHETE	**	****	n.s.
		8,15-DiHETE	*	***	n.s.
	**Isoprostanes**	5-$iPF_{2α}$	***	****	n.s.
		8-i-$PGF_{2α}$	****	****	**
	**Others**	5-oxo-ETE	n.s.	**	n.s.
		15-oxo-ETE	****	****	*
		11,12-,15-TriHETrE	*	****	*
		THF diol[a]	-	-	-
**EPA**	**Prostanoids**	PGE_3	***	****	****
		PGD_3	***	****	***
		d17-6k-$PGF_{1α}$	***	****	****
		TxB_3	****	****	****

	**Epoxy-FA**	8(9)-EpETE[d]	-	-	***
		11(12)-EpETE	*	****	****
		14(15)-EpETE	*	****	****
		17(18)-EpETE	n.s.	n.s.	n.s.
	**Dihydroxy-FA**	5,6-DiHETE[d]	**	-	-
		8,9-DiHETE[d]	-	-	****
		11,12-DiHETE[d]	-	-	****
		14,15-DiHETE[d]	-	-	****
		17,18-DiHETE	n.s.	n.s.	n.s.
	**Hydroxy-FA**	5-HEPE	n.s.	****	****
		8-HEPE[d]	-	-	*
		12-HEPE	*	****	**
		15-HEPE	****	****	***
		18-HEPE	**	****	****
		19-HEPE[d]	-	-	***
		20-HEPE	n.s.	*	n.s.
	**Multiple Hydroxylated FA**	LTB_5[c]	-	-	-
		RvE1[a]	-	-	-
		RvE2[a]	-	-	-
		18(S)-RvE3[a]	-	-	-
		18(R)-RvE3[a]	-	-	-
	**Others**	12-OH-17(18)-EpETE[a]	-	-	-
**DHA**	**Epoxy-FA**	10(11)-EpDPE	n.s.	****	****
		13(14)-EpDPE	n.s.	****	****
		16(17)-EpDPE	n.s.	****	****
		19(20)-EpDPE	n.s.	****	****
	**Dihydroxy-FA**	4,5-DiHDPE[d]	-	-	****
		7,8-DiHDPE[c]	-	-	-
		10,11-DiHDPE	n.s.	****	****
		13,14-DiHDPE	n.s.	****	****
		16,17-DiHDPE	***	****	***
		19,20-DiHDPE	*	****	****
	**Hydroxy-FA**	4-HDHA	n.s.	****	****
		7-HDHA	n.s.	****	****
		8-HDHA	n.s.	****	****
		10-HDHA	n.s.	*	n.s.
		11-HDHA	n.s.	**	**
		13-HDHA	*	****	**
		14-HDHA	n.s.	n.s.	n.s.
		16-HDHA	*	****	****
		17-HDHA	n.s.	n.s.	n.s.
		20-HDHA	*	****	****
		21-HDHA	n.s.	***	***
		22-HDHA	n.s.	***	**
	**Multiple Hy-droxylated FA**	RvD1[a]	-	-	-

**LA**	**Epoxy-FA**	9(10)-EpOME	n.s.	n.s.	n.s.
		12(13)-EpOME	n.s.	n.s.	n.s.
	**Dihydroxy-FA**	9,10-DiHOME	n.s.	n.s.	n.s.
		12,13-DiHOME	n.s.	n.s.	n.s.
	**Hydroxy-FA**	9-HODE	n.s.	*	*
		13-HODE	n.s.	*	n.s.
	**Others**	EKODE	n.s.	n.s.	n.s.
		9-oxo-ODE	n.s.	n.s.	n.s.
		13-oxo-ODE	n.s.	n.s.	n.s.
		9,12,13-TriHOME	n.s.	**	n.s.
		9,10,13-TriHOME	*	n.s.	n.s.
**ALA**	**Epoxy-FA**	9(10)-EpODE	n.s.	n.s.	n.s.
		12(13)-EpODE	n.s.	n.s.	n.s.
		15(16)-EpODE	n.s.	n.s.	n.s.
	**Dihydroxy-FA**	9,10-DiHODE	n.s.	n.s.	n.s.
		12,13-DiHODE[a]	-	-	-
		15,16-DiHODE	n.s.	n.s.	n.s.
	**Hydroxy-FA**	9-HOTrE	n.s.	n.s.	n.s.
		13-HOTrE	n.s.	n.s.	n.s.
**DGLA**	**Prostanoids**	PGE_1	****	****	n.s.
		PGD_1	***	****	n.s.
		$PGF_{1\alpha}$	**	***	n.s.
		15-k-$PGF_{1\alpha}$[a]	-	-	-
		13,14-dih-15-k-PGE_1	***	****	n.s.
		TxB_1	*	*	n.s.
	**Hydroxy-FA**	15(S)-HETrE	***	***	n.s.
**Oleic Acid**		9(10)-ep-stearic acid	n.s.	n.s.	n.s.
		9,10-dih-stearic acid	n.s.	*	**
**C20:3 n9**		LTB_3[a]	-	-	-
**C22:4 n6**		dihomo-$PGF_{2\alpha}$	***	****	*

[a] analyte below LOQ in all groups; [b] analyte below LOQ in WT-STD+n3 and *fat-1*; [c] analyte below LOQ in WT-STD and *fat-1*; [d] For analytes that were below LOQ one of the three groups, a t-test (two-tailed, unpaired), with Welch correction in case of unequal variances, was performed.
